# 16th European College of Equine Internal Medicine (ECEIM) Congress

**DOI:** 10.1111/jvim.17022

**Published:** 2024-02-21

**Lyon, France**

**October 27 to 28, 2023**

*The European College of Equine Internal Medicine (ECEIM) Congress and the Journal of Veterinary Internal Medicine (JVIM) are not responsible for the content or dosage recommendations in the abstracts. The abstracts are not peer reviewed before publication. The opinions expressed in the abstracts are those of the author(s) and may not represent the views or position of the ECEIM. The authors are solely responsible for the content of the abstracts*.

ORAL ABSTRACTS

- Retrospective case‐control study based on magnetic resonance imaging reveals asymmetry of the trigeminal nerve in horses with headshaking
  - F. Heun, Germany
- Detection of EqHV RNA in *Stomoxys calcitrans* in eastern Austria
  - V. Frisch, Austria
- The role of insulin clearance in hyperinsulinemia and its association with non‐alcoholic fatty liver disease in insulin‐dysregulated horses: A preliminary study
  - M. Dosi, UK
- Retrospective analysis of equine sarcoid treatment protocol and factors associated with recurrence
  - K.S. Offer, UK
- Infections with extended spectrum beta‐lactamase producing enterobacterales in hospitalized neonatal foals: Association with rectal colonization, risk factors and outcomes
  - A.S.T. Shnaiderman‐Torban, Israel
- Use of a point prevalence survey to measure antibiotic use in equine veterinary hospitals
  - E.K. Leus, South Africa
- Comparison of tracheal wash and bronchoalveolar lavage for cytological diagnosis of exercise‐induced pulmonary hemorrhage in horses
  - P. Barbazanges, France
- Long‐term follow‐up of colic recurrence and athletic performance in horses after explorative celiotomy
  - A.C. Calewaert, Belgium
- Activin A concentrations as a potential biomarker for detecting insulin dysregulation and predicting laminitis risk in ponies
  - C.J. McGuire, UK
- Quantification of immune cell populations in equine intestinal biopsies of healthy horses
  - M. Robel, Switzerland
- 8‐Hydroxy‐2′‐deoxyguanosine as a potential marker of oxidative damage in horses with neuroaxonal degeneration
  - M. Palmisano, USA
- Influence of N‐butylscopolammonium bromide and metamizol on echocardiographic variables in Warmblood horses with aortic and mitral valve regurgitation
  - A. Dufourni, Belgium
- Comparison of sequential versus average R–R intervals for evaluation of the degree of prematurity and pause length in arrhythmias in adult horses at rest
  - E. Williams Louie, USA
- Study into an improved Einthoven's triangle around the equine heart: proposition of the Delta (Δ) configuration.
  - E.P. Paulussen, Belgium
- Epidemiology of equine herpesvirus 1 myeloencephalopathy outbreak in Valencia 2021.
  - E.J.C. Jose‐Cunilleras, Spain
- Study of the survival of equine herpesvirus 4 (EHV‐4) as a source of environmental contamination
  - C. Normand, France
- Meta‐analysis of naturally occurring equine herpesvirus‐1 (EHV‐1) outbreaks finds no evidence for a significant effect of vaccination on the effective reproduction number.
  - R.M.A.C. Houben, The Netherlands
- Efficacy of an active vaccination against il‐5 for the treatment of equine recurrent urticaria
  - K. Birkmann, Switzerland
- The hypothalamic‐pituitary‐adrenal gland axis response to vasopressin (AVP) stimulation test in healthy and critically ill foals
  - K.A. Dembek, USA
- Increased serum thymidine kinase 1 activity is mostly not diagnostic for equine malignant lymphoma
  - K.A. Roscher, Germany
- Equine asthma severity correlates with neutrophil extracellular traps in bronchoalveolar lavage fluid
  - L.K. Meiseberg, Germany
- A new scoring system to evaluate mucosal surface pathology in equine glandular gastric disease
  - V.H.L Scott, UK
- Secretomic profiles to distinguish adult horses with non‐complicated acute gastro‐intestinal disease from those with sepsis
  - A.A. Leroux, France

POSTER ABSTRACTS

- A prospective, randomized study into the complications and outcomes of upper or lower eyelid subpalpebral lavage treatment systems in 66 equine eyes (2015‐2023)
  - A.E. Graham, UK
- Single‐cell transcriptome profiling of bronchoalveolar cells identifies a Th17 signature in severe equine asthma
  - S.E. Sage, Switzerland
- Immunohistochemical expression of cyclooxygenase‐2 (COX‐2) in equine melanomas
  - J. Pimenta, Portugal
- Determination of a mathematical score of survival in newborn foals: Retrospective study on foals admitted to intensive care at the Lyon Equine Vet Hospital (Clinéquine) between 2007 and 2020
  - A.A. Amar, France
- Effect of dopamine on glucose‐stimulated insulin production in the equine pancreas in vitro
  - M. Sillense, Australia
- Obesity and associated metabolic disease conditions in connemara ponies in Ireland
  - A. Al Ansari, Ireland
- Breath Characteristics and Adventitious Lung Sounds in Healthy and Asthmatic Horses: Assessment of a novel digital auscultation‐based method in equine asthma
  - E. Greim, Switzerland
- Neonatal piroplasmosis, an underestimated problem?
  - L.M.H. Hermans, France
- Salmonella shedding among colic cases presenting to an equine referral hospital in Qatar
  - M.R. Robin, Qatar
- Evaluation of symmetric (SDMA) and asymmetric (ADMA) dimethylarginines in healthy and in systemic inflammatory response syndrome (SIRS) negative or positive colic horse
  - F. Bindi, Italy
- Efficacy of intramuscular omeprazole in horses with ESGD and EGGD, with or without IBD, previously treated unsuccessfully with oral omeprazole
  - L.C. Kranenburg, The Netherlands
- Evaluation of a new smartphone‐based digital stethoscope featuring phonocardiography and electrocardiography in adult horses
  - F. Bindi, Italy
- Prevalence and role of gluten intolerance in 52 horses suspected of Inflammatory Bowel Disease
  - L.C. Kranenburg, The Netherlands
- The effects of detomidine infusion with and without vatinoxan on blood glucose and insulin concentrations in horses
  - N. Jantunen, Finland
- Ureterolithiasis in equids: A retrospective study of 7 cases (2013‐2022)
  - D. Jean, Canada
- Clinical findings of eosinophilic keratoconjunctivitis in horses
  - Z. Bakos, Hungary
- The effect of high‐carbohydrate feeding and body condition on pancreatic histomorphometry in mixed‐breed ponies
  - K. Timko, USA
- Contact force‐guided 3D electro‐anatomical mapping and radiofrequency ablation (CARTO3) for improved diagnosis and treatment of sustained atrial tachycardia in 9 horses
  - E. Buschmann, Belgium
- Dust generation and microbiological air quality with different bedding materials in a horse stables
  - C.U.P. Herholz, Switzerland
- Association between fungal detection and diagnosis of moderate equine asthma (mea) according to sampling site and methodology
  - P. Barbazanges, France
- Improvement of gastric ulcer and ridden horse pain ethogram scores with diet adaptation in sport horses
  - V.G.M. Pineau, Belgium
- Serial investigation of seroprevalence and fecal shedding of Lawsonia intracellularis in Thoroughbred foals in the first year of life
  - C. Ribonnet, UK
- Effects of tight nosebands on the upper airways of horses
  - D. Scholler, Germany
- Dynamics of training and acute exercise‐induced shifts in muscular glucose transporter (GLUT) 4, 8 and 12 expression in locomotion (M. vastus lateralis) versus posture muscles (M. pectoralis profundus) in healthy horses
  - C. Vidal de Moreno de Vega, Belgium
- NT‐proBNP as a potential biomarker for diagnosis, prognosis and monitoring of cardiac disease in horses
  - M. Demeyere, Belgium
- Baselining physiological parameters in posture versus locomotion muscles across breeds. Towards tailored dietary and training management
  - C. Vidal Moreno de Vega, Belgium
- Evaluation of the delta neutrophil index (DNI) in equine neonatal sepsis
  - N. Ellero, Italy

## RETROSPECTIVE CASE CONTROL STUDY BASED ON MAGNETIC RESONANCE IMAGING REVEALS ASYMMETRY OF THE TRIGEMINAL NERVE IN HORSES WITH HEADSHAKING

### **F. Heun**, J. Delarocque, M. Hellige, K. Feige

#### Clinic for Horses, University of Veterinary Medicine Hannover, Hannover, Germany

**Introduction**: Idiopathic trigeminal‐mediated headshaking (ITMHS) in horses may be associated with trigeminal nerve (TN) neuropathy. In human medicine, TN asymmetry was revealed by magnetic resonance imaging (MRI) correlating with nerve atrophy.

**Methods**: MRI examinations of 20 adult horses diagnosed with ITMHS were retrospectively compared to six horses that underwent MRI of the head for other reasons. All horses presented to the equine hospital of the University of Veterinary Medicine Hannover, Foundation in 2021 and 2022. MRI studies were obtained with a 3 Tesla MRI scanner (Philips Achieva 3T dStream). Standardized cross‐sectional area measurements of branches of the TN were performed bilaterally at 4 defined locations (1: rostral to the trigeminal ganglion, 2: close to the pituitary gland, 3: close to the optic chiasm, 4: rostral to the foramen rotundum). Bilateral cross‐sectional transverse area differences (TN asymmetry) were calculated and compared between the two groups using a generalized additive model of the Tweedie family.

**Results**: Horses with ITMHS had 2 to 8.7 times higher TN asymmetry than controls (*F*[1] = 22.093, *P* < .001). The difference in TN asymmetry was most pronounced rostral to the trigeminal ganglion—area 1 (back‐transformed estimated marginal mean ratio and SE: 8.66 ± 3.975, *t*[212] = 4.700, *P* < .0001). There was no effect of age of the horse or duration of ITMHS on the TN asymmetry.

**Discussion and Clinical Relevance**: The asymmetry of the TN in horses with ITMHS indicates a unilateral enhanced neuropathy similar to findings in humans. Few previous studies provided evidence of atrophy or dysfunction of the TN in horses diagnosed with ITMHS, moreover, this MRI study revealed morphological changes of the TN in horse and provides directions for further studies and diagnostic approaches.

## DETECTION OF EqHV RNA IN STOMOXYS CALCITRANS IN EASTERN AUSTRIA

### **V. Frisch** ^1^, S. Ramsauer^2^, I. Preining^1^, M.S. Unterköfler^1^, T. Borysova^1^, M. Haller^1^, J. Wigger^1^, H.‐P. Führer^1^, J.‐M.V. Cavalleri^1^

#### ^1^University of Veterinary Medicine Vienna, Vienna, Austria; ^2^University of Zurich, Zurich, Switzerland

**Introduction**: The global prevalence of Equine Hepacivirus (EqHV), a member of the family *Flaviviridae*, is discussed to range from 3.6% to 16.1%. The EqHV antibody prevalence in eastern Austria in the horse population is high with 45.9%. These numbers suggest a horizontal way of transmission, however only vertical and iatrogenic viral transmission were detected to date. Mechanical transmission routes by vectors are discussed, but have not been thoroughly investigated. This is the first study investigating the detection of EqHV RNA in *Stomoxys calcitrans* (stable fly), a member of the family Brachycera.

**Methods**: In 2021, 606 *Stomoxys calcitrans* were caught in two horse stables in the surroundings of Vienna and at the University Equine Hospital of the Veterinary University Vienna. Maximum 5 flies' heads and thoraxes including legs and wings were pooled and analyzed for the presence of EqHV RNA by reverse transcription quantitative polymerase chain reaction (RT‐qPCR). The minimum infection rate (MIR) was calculated ([number of positive pools/total number of flies tested] × 100), to determine the infection rate of the analyzed stable flies.

**Results**: In 7 out of 135 pools, collected in September at one stable in eastern Austria, EqHV RNA could be detected. The MIR of *Stomoxys calcitrans* in 2021 was 1.2%. The viral RNA was detected in females as well as in male flies.

**Conclusions**: This is the first study detecting the presence of EqHV RNA in *Stomoxys calcitrans* and reporting a potential, possibly mechanical vector for this virus.

**Clinical Relevance**: The detection of EqHV RNA in *Stomoxys calcitrans* suggests a possible horizontal way of transmission. Action concerning husbandry management against viral infection can only be initiated after identification of transmission routes.

## THE ROLE OF INSULIN CLEARANCE IN HYPERINSULINEMIA AND ITS ASSOCIATION WITH NON‐ALCOHOLIC FATTY LIVER DISEASE IN INSULIN‐DYSREGULATED HORSES: A PRELIMINARY STUDY

### R. Ruth ^1^, H. Holly^1^, L. Scott^1^, **M. Dosi** ^2^, J.A. Keen^2^, B. McGorum^2^, A. Alexandra^2^

#### ^1^Scotland Rural College, Edinburgh, UK; ^2^University of Edinburgh, Edinburgh, UK

**Introduction**: Horses with endocrinopathies often have sustained hyperinsulinemia. In humans, reduction of insulin hepatic clearance participates to the hyperinsulinemia and has been associated with non‐alcoholic fatty liver disease (NAFLD), although this is not known in horses.

**Methods**: Tissue samples were collected at post‐mortem from horses with and without (n = 14) hyperinsulinemia associated with EMS (n = 10) or PPID (n = 10). Healthy horses were euthanized for unrelated diseases and included if they did not have liver pathologies. Equine Metabolic Syndrome (EMS) was defined as a body condition score >3.5/5, fasting basal insulin >20 mL U/L, history of laminitis. Pituitary Pars Intermedia Dysfunction (PPID) was defined according to ACTH seasonal cut‐off, and histological changes to the pituitary. Liver tissue was fixed in 10% formalin, paraffin‐embedded and 5 μm sections stained with hematoxylin and eosin (H&E) and immunohistochemically for CEACAM1, an insulin degrading protein. Each section was scored by two blinded observers using the equine liver disease scoring system (Durham et al 2003) and a human NAFLD scoring system (Bedossa et al. 2012). In frozen liver sections, triglycerides and Insulin Degrading Enzyme (IDE) activity were quantified.

**Results**: Although the cumulative NAFLD scores and hepatic triglyceride content were not significantly different between groups, the most severe changes were seen in horses with endocrine disease (3/20). There was no correlation between NAFLD score and basal insulin measurement. CEACAM1 was identified in all horses by immunohistochemistry, but the study was underpowered to show differences between groups. Quantitative tests are required to confirm these results. IDE activity was significantly decreased in horses with hyperinsulinemia compared with controls (controls 3 IQR 4.50, EMS 2.18 IQR 1.57 activity/mg protein).

**Discussion**: While NAFLD is not a feature of equine hyperinsulinemia, there are differences in insulin clearance proteins suggesting that reduced clearance is likely a contributing factor and could represent a therapeutic target.

## RETROSPECTIVE ANALYSIS OF EQUINE SARCOID TREATMENT PROTOCOL AND FACTORS ASSOCIATED WITH RECURRENCE

### **K.S. Offer**, D.G.M. Sutton

#### University of Glasgow, Glasgow, UK

**Introduction**: Treatment methods reported for equine sarcoids (ES) include laser excision and cryotherapy. Concurrent 5‐fluorouracil (5‐FU) may aid in cell death at the periphery of lesions treated with cryotherapy. This study aimed to compare laser excision with a combination of laser excision, cryotherapy, and 5‐FU chemotherapy for ES treatment. Factors associated with sarcoid recurrence were also investigated.

**Methods**: Medical records were reviewed for horses treated at Glasgow University for ES using the above two protocols (2013‐2022). Inclusion required histological confirmation of sarcoids. Treatment outcome was determined by owner communication. Univariate and multinominal logistic regression was performed to identify variables associated with recurrence. Cox's proportional hazards model was employed to identify variables influencing time to recurrence.

**Results**: Exactly 84 horses and 168 sarcoids were suitable for inclusion. Overall recurrence rate was 23%. Previous sarcoid treatment (OR 7.6 [2.0‐33]), individual sarcoid diameter ≥100 mm (OR 5.6 [1.1‐30]), requirement for general anesthesia (OR 5.0 [1.4‐19]) and total number of sarcoids (OR 1.2 [1.0‐1.5]) were associated positively with recurrence, in contrast to confirmation of surgical margins (OR 0.40 [0.005‐2.3]). For time to sarcoid recurrence, lower limb (HR 0.2 [0‐1.6]) and first ES episode (HR 0.3 [0.1‐0.7]) decreased risk, which was increased by ≥1 mixed sarcoids (HR 9.9 [3.3‐30]) and urogenital location (HR 3.6 [1.3‐10]). Treatment category was not associated with either sarcoid recurrence or time to recurrence.

**Conclusions**: In this retrospective study, the individual characteristics of the sarcoid, number and location of lesions and previous treatment attempts were of greater influence on recurrence rate than individual treatment modality.

**Clinical Relevance**: Sarcoid masses should be treated at the first opportunity, with particular attention to surgical margins. Mixed sarcoids affecting the urogenital region merit increased care. Multimodal treatment may be of benefit in ES treatment, but prospective intervention studies are required to determine efficacy.

## INFECTIONS WITH EXTENDED SPECTRUM BETA‐LACTAMASE PRODUCING ENTEROBACTERALES IN HOSPITALIZED NEONATAL FOALS: ASSOCIATION WITH RECTAL COLONIZATION, RISK FACTORS AND OUTCOMES

### **A.S.T. Shnaiderman‐Torban** ^1^, L.M. Meltzer^2^, T.Z.D. Zilberman Daniels, S.N.V. Navon‐Venezia^3^, G.A.S. Abells Sutton^1^, A.K. Kohen^1^, S.B. Blum^4^, S.A. Amit^2^, A.S. Steinman^1^

#### ^1^KSVM Israel, Rehovot, Israel; ^2^Sheba Medical Center, Ramat Gan, Israel; ^3^Ariel University, Ariel, Israel; ^4^Kimron Veterinary Institute, Rishon LeZion, Israel

**Introduction**: Infections with extended spectrum beta lactamase producing *Enterobacterales* (ESBL‐PE) contribute to morbidity and mortality among newborn human infants. In equine neonatal medicine, data is scarce.

**Methods**: This prospective, single center cohort study, assessed 67 neonatal foals presented to a veterinary teaching hospital. Foals were screened for ESBL‐PE rectal colonization on admission. From admission onwards, bacterial isolates from blood, umbilicus, IV catheter and joint were tested for ESBL production. Medical data were analyzed for risk factors and clinical outcomes.

**Results**: The prevalence of ESBL‐PE colonization and infections were 46% (n = 31/67) and 52% (n = 35/67), respectively. Colonization on admission was significantly associated with an ESBL‐PE infection during hospitalization (*P* = .018). On multivariable logistic regression analysis, colonization on admission was associated with the Arabian breed (*P* = .014, OR = 14.5). Clinical signs of an umbilical infection on admission were associated with an ESBL‐PE infection during hospitalization (OR = 4.8, *P* = .004). In an outcome analysis, an ESBL‐PE infection was associated with surgery during hospitalization (OR = 5.2, *P* < .001) and with a longer length of stay (OR = 8.1, *P* < .001).

The main ESBL‐PE species isolated from both rectal screening and clinical samples was *Escherichia coli* (52%, n = 27/52 and 44%, n = 18/41, respectively). Concordant ESBL‐PE species was recovered from a rectal screening sample and at least one clinical sample in 16% of foals (n = 11/67).

**Conclusions**: On‐admission ESBL‐PE rectal colonization is associated with ESBL‐PE infections in neonatal foals. Such infections are related to surgery during hospitalization and a longer length of stay.

**Clinical Relevance**: This study demonstrates an alarming prevalence of ESBL‐PE colonization and infection, and the significance of rectal screening on admission as a predictor for infection with ESBL‐PE during hospitalization.

## USE OF A POINT PREVALENCE SURVEY TO MEASURE ANTIBIOTIC USE IN EQUINE VETERINARY HOSPITALS

### **E. K. Leus** ^1^, C.H. Lyle^1^, N. Collins^2^, M. Gruyaert^3^, R.N. Kennedy^1^, E. McConnell^4^, B.C. McGorum^5^, D. Luethy^6^, M. Sanz^7^, A. Versporten^8^, A. Viljoen^9^

#### ^1^Vetscape Equine Referrals, Paarl, South Africa; ^2^Scone Equine Hospital, Scone, Australia; ^3^Faculty of veterinary medicine Ghent university, Merelbeke, Belgium; ^4^Murdoch University, Perth, Australia; ^5^Edinburgh University, Edinburgh, UK; ^6^University of Florida, Gainesville, USA; ^7^Washington State University, Washington, USA; ^8^University of Antwerp, Antwerp, Belgium; ^9^University of Pretoria, Pretoria, South Africa

**Introduction**: Antibiotic resistance is increasingly recognized in equine medicine. Antibiotic use is a key driver of antibiotic resistance. This study piloted a point prevalence survey (PPS), based on the Global‐PPS used in human hospitals, to obtain data on antibiotic prescribing in equine hospitals. The aim was to determine if the PPS could be used as an antibiotic stewardship tool to identify targets for improvement in antibiotic use.

**Methods**: Eight equine hospitals located in Australia, Belgium, South Africa, the United Kingdom and the United States of America were recruited. Data on antibiotic use were collected from all in‐patients on antibiotic treatment at 8 am on four selected study days throughout the study year (2022). Data collected included patient details, antibiotics prescribed, indication, use of a treatment stop/review date and adherence to local guidelines.

**Results**: A total of 742 patients, 310 (41.8%) surgical and 432 (58.2%) nonsurgical cases, were evaluated. A total of 58.7% of the surgical patients were on antibiotic treatment, compared to 25.9% of the nonsurgical patients. The most prescribed antibiotics were penicillin, gentamicin, and trimethoprim sulphonamides. In 45.2% of treatments, use was prophylactic (33.2% surgical prophylaxis and 12% nonsurgical prophylaxis). Therapeutic use was based on a biomarker in 48% of the treatments. A sample was submitted for culture in 56.9% of therapeutic treatments. An antibiotic use review/stop date was not recorded in 59.5% of treatments. Local guidelines did not exist for 27.5% of treatments.

**Conclusions and Clinical Relevance**: The PPS identified multiple ways in which antibiotic use could be optimized. Targets identified for stewardship interventions included the high prevalence of prophylactic use and the lack of use of a stop/review date. The survey could be used as a repeatable tool to assess the impact of stewardship interventions in equine hospitals.

## COMPARISON OF TRACHEAL WASH AND BRONCHOALVEOLAR LAVAGE FOR CYTOLOGICAL DIAGNOSIS OF EXERCISE‐INDUCED PULMONARY HEMORRHAGE IN HORSES

### **P. Barbazanges** ^1^, E.A. Richard^2^, L.C. Lemonnier^1^, M.P. Toquet^2^, A. Couroucé^1^

#### ^1^Oniris, Nantes, France; ^2^LABEO, Saint‐Contest, France

**Introduction**: Various methods are reported for diagnosis of exercise‐induced pulmonary hemorrhage (EIPH). Cytological evaluation of airway samples is a sensitive method, but the correlation between tracheal wash (TW) and bronchoalveolar lavage fluid (BALF) findings for diagnosis of EIPH is unknown.

The objective was to determine whether diagnosis of EIPH, using hemosiderophages/macrophages (H/M) ratio, differs when based on samples from TW and BALF collected concomitantly from the same racehorse.

**Methods**: Prospective cross‐sectional study on 102 Standardbred horses in active training. TW and BALF from each lung separately were collected from all horses at rest. Smears were stained with May‐Grünwald‐Giemsa (MGG) and H/M ratio calculated. Diagnostic cut‐off values were set at 17% for individual (left and right) BALF and 9% for pooled BALF. H/M ratio in TW samples were scored as none (0%), occasional (<10%), small (10%‐25%), moderate (25%‐50%) or large proportions (>50%).

**Results**: In BALF, 21 horses met the cytological inclusion criteria for EIPH diagnosis from individual and/or pooled samples. In TW, 20 horses had occasional proportions of hemosiderophages, and respectively 9, 1 and 3 horses had small, moderate and large proportions. Poor correlations between TW and respectively pooled, left and right BALF were found for H/M ratio. Among the 13 horses with at least small proportions of hemosiderophages in TW, 8 (61.5%) had no cytological evidence of EIPH in any BALF.

**Conclusion and Clinical Relevance**: No association between TW and BALF was found for the cytological diagnosis of EIPH. A large number of horses has cytological evidence of pulmonary bleeding in BALF with none or occasional proportions of hemosiderophages in TW. In addition, finding small to large proportions of hemosiderophages in TW is mostly not associated with evidence of pulmonary hemorrhage in BALF.

Based on H/M ratio, BALF remains the sample of choice for cytological diagnosis of EIPH.

## LONG‐TERM FOLLOW‐UP OF COLIC RECURRENCE AND ATHLETIC PERFORMANCE IN HORSES AFTER EXPLORATIVE CELIOTOMY

### A.C. Calewaert

#### Ghent University, Merelbeke, Belgium

**Introduction**: Exploratory celiotomy is a critical and expensive intervention for treating colic in horses. Insight into the long‐term effect on athletic performance and recurrence of colic is important for decision‐making.

**Methods**: An email survey was conducted and answered by 96 horse owners whose horses had undergone colic surgery at Ghent University between 2013 and 2019. The questionnaire aimed to gather long‐term (>3 years) follow‐up information, focusing on predisposition to new colic episodes and changes in athletic performance after the surgery.

**Results**: The majority (72%) of horses were still owned by their previous owners, while 13% had been sold. 15% of the horses had died, out of which 78% causes of death were related to a recurrent colic episode. Among all the horses included in this study, 42% had initial small intestinal colic, while 58% had initial large intestinal colic. According to the owners' reports, 10% of horses with initial small intestinal colic and 21% of horses with initial large intestinal colic seemed more prone to redeveloping colic signs. Regarding athletic performance, owners reported that 89% of the horses maintained or even increased their pre‐surgery performance level, 5% decreased the level of performance due to the owners' decision and 12% performed at a lower level. The latter group consisted of 30% small intestinal and 70% large intestinal colic cases.

**Conclusion and Clinical Relevance**: Based on the owners' perception, only a small percentage of horses exhibited a renewed colic episode following surgery. Most of the horses were able to maintain or even improve their performance levels. These findings emphasize the positive long‐term outcomes for athletic performance after an exploratory celiotomy in horses with colic.

## ACTIVIN A CONCENTRATIONS AS A POTENTIAL BIOMARKER FOR DETECTING INSULIN DYSREGULATION AND PREDICTING LAMINITIS RISK IN PONIES

### **C.J. McGuire** ^1^, E.J. Knowles^1^, P.A. Harris^2^, N.J. Menzies‐Gow^1^

#### ^1^Royal Veterinary College, Hertfordshire, UK; ^2^Waltham Petcare Science Institute, Leics, UK

**Introduction**: Basal plasma Activin A (AA) concentration has recently been shown to positively correlate with insulin concentrations 60 minutes following an oral sugar test (OST) in ponies with equine metabolic syndrome, therefore there is potential for this to be used as a marker for insulin dysregulation. Insulin concentrations at baseline (T0) or 60 minutes (T60) post OST best quantify the risk of future laminitis in non‐laminitic ponies. The study aim was to explore the relationship between AA concentrations at T0 and T60, in addition to other metabolic markers in ponies that subsequently develop laminitis and those that remain non‐laminitic.

**Methods**: Case‐control study design. Forty‐two ponies that developed laminitis during a 4‐year surveillance period were selected from a cohort of 374 non‐laminitic animals; 42 ponies from the same cohort that remained non‐laminitic were selected as controls. Plasma AA concentrations were measured using a validated ELISA at T0 and T60 post OST. Serum insulin, blood glucose and plasma triglyceride, adrenocorticotrophin hormone and adiponectin concentrations had been previously measured. Plasma AA concentrations were compared between groups at T0 and T60 using Kruskal‐Wallis one‐way ANOVA and correlations with other metabolic markers investigated using Spearman correlations.

**Results**: There was no significant difference between Plasma AA and Insulin concentrations at either time point, nor between Plasma AA concentrations and any other metabolic markers or pony parameters between the two pony groups. There was a significant positive correlation between Plasma AA T0 and T60 (*P* < .001, *r* ^2^ = .67) and a significant positive correlation between the increase in Plasma AA and the increase in insulin concentrations between T0 and T60 in the control ponies (*P* = .009, *r* ^2^ = .399), but not the laminitic pony group.

**Clinical Relevance**: Plasma AA concentration is not a useful marker for ID and does not appear to be associated with laminitis development.

## QUANTIFICATION OF IMMUNE CELL POPULATIONS IN EQUINE INTESTINAL BIOPSIES OF HEALTHY HORSES

### **M. Robel** ^1^, P. Grest^1^, A. Schoster^2^

#### ^1^University of Zurich, Zurich, Switzerland; ^2^Ludwig‐Maximilians‐University Munich, Oberschleissheim, Germany

**Introduction**: There are little data on normal inflammatory cell counts (ICCs) in the intestinal wall of healthy horses. Often only minimally invasive obtained mucosal biopsies are available for evaluation. The objective was to describe and compare inflammatory cells in different locations and biopsy types of healthy horses.

**Methods**: Full‐thickness and endoscopic forceps obtained mucosal biopsies were taken post‐mortem from the duodenum and rectum of 15 healthy horses slaughtered for meat production. After fixation in 10% buffered formalin, samples were stained with hematoxylin and eosin (HE) and immunohistochemistry (IHC; CD3, CD20, Iba‐1) was performed. Four‐ten fields with 0.01 mm^2^ or 250 to 500 cells or 4 to 10 High Power fields were evaluated per sample. ICCs between biopsy types and locations were compared with the Steel‐Dwass test.

**Results**: Depending on biopsy type, median lymphocyte counts in the epithelium and lamina propria of the duodenum (LP) on HE stains were 4.9 to 9 and 3.8 to 8.5 and on IHC_CD3_ 7.6 to 15.2 and 7.35 to 15. In the duodenum ICCs were significantly higher in the epithelium and LP of full‐thickness biopsies compared to mucosal biopsies (all *P* < .04) except for macrophages (*P* = .3). There was no difference in ICC between biopsy types in the rectum (all *P* > 0.3). In full‐thickness biopsies ICCs were significantly higher in the duodenum (Lamina propria (LP)‐plasma cells_HE_ *P* = .003, LP‐B‐cells_CD20_ *P* = .01). In mucosal biopsies LP‐eosinophils were significantly higher in the rectum (*P* = .005).

**Discussion**: Higher ICCs were present in full thickness biopsies of the duodenum, likely due to the greater biopsy depth. Higher ICCs were present in the duodenum, except for eosinophils which were higher in the rectum. IHC increased the detection of immune cells.

**Clinical Relevance**: ICCs need to be interpreted based on intestinal segment and biopsy type.

## 8‐HYDROXY‐2′‐DEOXYGUANOSINE AS A POTENTIAL MARKER OF OXIDATIVE DAMAGE IN HORSES WITH NEUROAXONAL DEGENERATION

### M. Palmisano

#### Large animal internal medicine department, University of Pennsylvania's New Bolton Center

**Background**: Equine neuroaxonal dystrophy/equine degenerative myeloencephalopathy (eNAD/EDM) is increasingly recognized as a cause of neurologic disease in adult horses. Diagnosis requires postmortem examination, with no accurate antemortem diagnostic test available. 8‐Hydroxy‐2′‐deoxyguanosine (8‐OHdG), a biomarker of oxidative damage utilized in human neurodegenerative disease, has potential to correlate with postmortem diagnosis of eNAD/EDM.

**Hypothesis/Objectives**: We hypothesized that 8‐OHdG will be increased in CSF and serum from eNAD/EDM horses compared to horses with other neurologic diseases and a control group of neurologically normal horses. 8‐OHdG will be increased in CSF compared to serum of eNAD/EDM horses.

**Animals**: Fifty client‐owned horses with postmortem diagnoses: 20 eNAD/EDM, 10 CVSM, 10 EPM, and 10 control horses. Serum and CSF samples were obtained from November 2010 through March 2022.

**Methods**: Case‐control study using biobanked samples was performed and commercial competitive ELISA kit (Highly Sensitive 8‐OHdG Check ELISA) utilized. Concentration of 8‐OHdG was quantitated in both CSF and serum and compared between groups.

**Results**: The median concentration of 8‐OHdG among all groups was 156.9 pg/mL (41.5‐635.4) in CSF and 125.3 pg/mL (36.8‐857.5) in serum. Poisson regression showed no significant difference (*P* > .05) once confounding variables (breed, age, sample age, vitamin E concentration) were considered.

**Conclusions**: 8‐OHdG is detectable in equine CSF and serum, although does not aid in antemortem diagnosis of eNAD/EDM based on this population of horses. The findings support that at the time of diagnosis horses with eNAD/EDM do not have ongoing oxidative stress. Further studies are needed to improve our ability to obtain an antemortem diagnosis.

## INFLUENCE OF N‐BUTYLSCOPOLAMMONIUM BROMIDE AND METAMIZOL ON ECHOCARDIOGRAPHIC VARIABLES IN WARMBLOOD HORSES WITH AORTIC AND MITRAL VALVE REGURGITATION

### **A. Dufourni**, M. Demeyere, G. Loon, A. Decloedt

#### Ghent University, Merelbeke, Belgium

**Introduction**: N‐butylscopolammonium bromide (NBB) causes transient tachycardia and hypertension, which has anecdotally been associated with intensified cardiac murmurs. Administration of Buscopan compositum (NBB and metamizol sodium) might be useful as a pharmacological stress test for evaluating valvular regurgitation. We hypothesized that the regurgitant jet area increases with higher heart rates and blood pressure.

**Methods**: Regurgitant jet areas, cardiac dimensions and function were measured in horses with aortic (AR, n = 10) and mitral valve regurgitation (MR, n = 10) by 2D‐, M‐mode and color Doppler echocardiography before and after intravenous administration of 0.2 mg/kg N‐butylscopolammonium bromide and 25 mg/kg metamizol sodium. Measurements were performed by a blinded observer. Data were analyzed using repeated measures analysis of variance.

**Results**: Compared to rest, Buscopan compositum administration resulted in similar increases in mean heart rate in horses with AR and MR (38 ± 5 vs 71 ± 12 bpm, *P* < .001). The regurgitant jet area increased in horses with AR and MR (*P* = .017). Left ventricular end‐diastolic area (174 ± 20 vs 148 ± 12 cm^2^, *P* = .002), volume (1435 ± 273 vs 1107 ± 158 mL, *P* = .004) and internal diameter on M‐mode (12.1 ± 0.9 vs 11.0 ± 1.3 cm, *P* = .036) decreased significantly in horses with AR, but not significantly in horses with MR. Left atrial fractional area change increased significantly in horses with AR (24% ± 10% vs 41% ± 6%, *P* = .001) and MR (23% ± 6% vs 31% ± 7%, *P* = .012).

**Conclusion**: Buscopan compositum administration results in an increased regurgitant jet area in horses with aortic and mitral valve regurgitation.

**Clinical Relevance**: Buscopan compositum exacerbated valvular regurgitation and may be useful as pharmacological stress test in horses with AR and MR. With a similar increase in heart rate, Buscopan resulted in a much more pronounced decrease in left ventricular dimensions in horses with AR compared to MR. This finding emphasizes the importance of heart rate while evaluating cardiac dimensions during follow‐up exams in horses with AR.

## COMPARISON OF SEQUENTIAL VERSUS AVERAGE R‐R INTERVALS FOR EVALUATION OF THE DEGREE OF PREMATURITY AND PAUSE LENGTH IN ARRHYTHMIAS IN ADULT HORSES AT REST

### **E. Williams Louie** ^1^, B. Suter^2^, C.C. Schwarzwald^2^, K. Mitchell^1^

#### ^1^Cornell University, Ithaca, USA; ^2^University of Zurich, Zurich, Switzerland

**Introduction**: Criteria for arrhythmia classification in horses is lacking. This study investigates the %R‐R interval change using comparison of sequential and average R‐R intervals in atrial (APC) and ventricular (VPC) premature complexes and sinus arrhythmia (SA).

**Methods**: Arrhythmias were classified as APC, VPC or SA, with 9 R‐R intervals recorded; the premature interval at position 4 and pause at position 5 in each sequence. The %R‐R deviation between sequential and average (intervals 1‐3) intervals were calculated. Comparison between arrhythmia classification and sequential vs average R‐R intervals were analyzed by ANOVA.

**Results**: Thirty ECGs were included; 10 each classified as APC, VPC and SA.

The sequential % prematurity was significantly different between APC vs VPC (mean diff: 95% CI; 17%: 3%‐32%, *P* = .013) and VPC vs SA (−28%: −42% to −13%, *P* < .0001). The sequential % pause was significantly different between all groups: APC vs VPC (−133%: −147% to −119%, *P* < .0001); VPC vs SA (178%: 164%‐192%, *P* < .0001); APC vs SA (45%: 31%‐60%, *P* < .0001).

When comparing to the average of R‐R intervals all groups were significantly different; in % prematurity, APC vs VPC (16%: 12%‐21%, *P* < .0001), VPC vs SA (−27%: −31% to −23%, *P* < .0001) and APC vs SA (−10%: −14% to −6%, *P* < .0001); and in the % pause, APC vs VPC (−34%: −38% to −30%, *P* < .0001), VPC vs SA (52%: 48%‐56%, *P* < .0001) and APC vs SA (18%: 14%‐22%, *P* < .0001).

**Discussion**: Comparing the average (intervals 1‐3) to the premature interval and subsequent pause offers greater differentiation between APCs, VPCs and SA than sequential R‐R intervals. More investigation using P‐P intervals in place of R‐R intervals and evaluating arrhythmias at exercise is needed.

**Clinical Relevance**: Sinus arrhythmia can be differentiated from APCs by the duration of the pause with both sequential and average R‐R interval analysis. Compared to APCs, VPCs are more premature with longer pauses.

## STUDY INTO AN IMPROVED EINTHOVEN'S TRIANGLE AROUND THE EQUINE HEART: PROPOSITION OF THE DELTA (Δ) CONFIGURATION

### **E.P. Paulussen** ^1^, G.V.S. Glenn^1^, A.D.C. Decloedt^1^, B.H. Hermans^2^, T.D. Delhaas^2^, G. van Loon^1^

#### ^1^Ghent University, Merelbeke, Belgium; ^2^Maastricht University, Maastricht, The Netherlands

**Introduction**: The absence of a standardized, data‐driven electrode configuration for equine electrocardiogram (ECG) recording hinders the advancement of arrhythmia diagnostics and data exchange between equine clinicians. Commonly used configurations are effective for rate and rhythm diagnosis but remain limited in identifying the origin of arrhythmias.

**Objectives**: The study aimed to assess various Einthoven's triangle electrode configurations to enhance ECG recordings in horses. Optimal ECG recordings are characterized by a large P and QRS amplitude, along with electrodes positioned on both sides of the body, to differentiate between left‐ and right‐sided ectopy. We hypothesized that a base down triangle (called Delta Δ configuration) would yield increased diagnostic information to fulfill these criteria.

**Animals**: Seventy‐five healthy warmblood horses aged 4 to 20 years were examined. Thirty electrodes were placed and a 5‐minute ECG recording at 1000 Hz sampling rate was made using a wearable physiological signal amplifier system.

**Methods**: We compared the modified base‐apex configuration with the Dubois, Copenhagen and our own proposed Δ configuration (Figure 1). A weighted score was calculated based upon the following normalized criteria: P and QRS amplitude, P/R ratio, Q/T ratio, and coherence. All signal processing was done with custom scripts in Matlab.

**Results**: The modified base‐apex configuration, which does not include left‐right information, scored 16.5 ± 3.5. For systems with Einthoven's triangle around the heart scores were 16.0 ± 3.6 for Δ, 13.7 ± 3.6 for Copenhagen, and 13.1 ± 2.8 for Dubois.

**Conclusions and Clinical Relevance**: The Δ configuration is promising because of sufficiently large P and QRS amplitudes, and best overall scores. Having the base of Einthoven's triangle along the ventral part of the heart is likely to result in better left‐right differentiation of ectopic rhythms and improve vectorcardiography results. Implementing this new configuration could lead to improved arrhythmia diagnosis in horses.

## EPIDEMIOLOGY OF EQUINE HERPESVIRUS 1 MYELOENCEPHALOPATHY OUTBREAK IN VALENCIA 2021

### **E.J.C. Jose‐Cunilleras** ^5^, M.C.T. Cuesta‐Torrado^1^, I.S.L. Santiago‐Llorente^2^, L.A.R. Armengou^3^, A. Velloso Alvarez^1^, F.N. Nieto^4^, J.R. Ríos^5^, F.C.L. Cruz‐Lopez^2^

#### ^1^Universidad Cardenal Herrera CEU, Valencia, Spain; ^2^Universidad Complutense, Madrid, Spain; ^3^Universitat Autonoma de Barcelona, Bellaterra, Barcelona, Spain; ^4^Equihealth Veterinarios, La Roca del Vallès, Barcelona, Spain; ^5^Universitat Autonoma de Barcelona, Bellaterra (Barcelona), Spain

**Introduction**: An EHV‐1 myeloencephalopathy (EHM) outbreak occurred in Valencia, Spain, in 2021. The aim of this study was to evaluate risk factors, effective reproduction rate (Rt) and long‐term outcomes of this severe outbreak.

**Methods**: Retrospective epidemiological study based on clinical records from the veterinary team at the competition site and three referral equine hospitals. All quarantined (n = 160) and referred (n = 31) horses were included in this study. All horses had at least one EHV‐1 PCR test performed in blood/nasal swabs. Association of risk factors (sex, vaccination, age, breed, viral load on PCR) with mortality, development of EHM, and odds of returning to competition by 2 years, were estimated by odds ratios [95% CI]. Rt was estimated by the Robert Koch's Institute method.

**Results**: Out of a total of 191 horses, 11 died or were euthanized, 78 developed EHM and survived, 50 were febrile, 14 were asymptomatic (positive PCR) and 38 remained healthy. Vaccination rate for EHV was 30%. There were 84 mares, 80 geldings, and 27 stallions. Previous EHV vaccination was associated with development of EHM (4.4 [2.2‐8.7]; *P* < .0001). Mares were over‐represented among nonsurviving horses: 8/11, 3/11, and 0/11 (mares, geldings, and stallions, respectively). The likelihood of returning to competition was similar in horses recovered from EHM (73%; 57/78) and infected horses without neurologic disease (76%; 47/62). Rt was 5.3 early on and decreased to 1.9 by 2 weeks from onset of outbreak.

**Conclusions**: Sex and vaccination status appeared to play a role in development of EHM or increase the risk of mortality after EHV1 infection. Horses recovered from EHM could be as likely to return to competition as other EHV‐1 infected horses.

**Clinical Relevance**: Further studies are necessary to establish the role of individual risk factors, vaccination and level of herd immunity on development of EHM.

## STUDY OF THE SURVIVAL OF EQUINE HERPESVIRUS 4 (EHV‐4) AS A SOURCE OF ENVIRONMENTAL CONTAMINATION

### **C. Normand** ^1^, A. Guenoux^2^, C. Fortier^2^, E. Hue^2^, P.H. Pitel^3^, S. Pronost^2^

#### ^1^LABÉO – BIOTARGEN, Saint‐Contest, France; ^2^LABÉO – BIOTARGEN, Saint‐Contest, France; ^3^LABÉO, Saint‐Contest, France

**Introduction**: Equine herpesvirus 4 (EHV‐4) is responsible for respiratory diseases, called equine viral rhinopneumonitis, in horses and in rare cases for abortions or neonatal deaths. This disease is highly contagious and transmitted mainly through infected nasal discharge. However, studies of the survival of EHV‐1 in water and on inanimate surfaces highlight a potential source of infection for susceptible horses.

**Methods**: Water was spiking with EHV‐4 and incubated at 4 temperatures (4°C, 20°C, 27°C, and 34°C) for up to 21 days (34°C) or 105 days (20°C, 27°C, and 34°C). The survival of EHV‐4 in water was analyzed by qPCR and an innovative culture cellular method: Real‐Time Cell Analysis. The detection of EHV‐4's genome in the environment was investigated during episodic EHV‐4 in stud farms.

**Results**: Depending on the temperature, EHV‐4 is able to remain infectious in water. For example, at 4°C, EHV‐4 is infective for up to a quarantine period (21 days). EHV‐4 was also detected on surfaces like troughs, feeders, metal grids, and others.

**Conclusions**: These results suggest that the environment could be an abiotic source of contamination by EHV‐4 for susceptible horses. Also, this study highlights the necessity to improve preventive strategies, through biosecurity measures (disinfection), during outbreaks.

**Clinical Relevance**: This work highlights the risk of environmental contamination, which can cause individual contamination leading to an epizootic. EHV‐4 and rhinopneumonitis provide a good model for studying the survival of equine viruses in the environment.

## META‐ANALYSIS OF NATURALLY OCCURRING EQUINE HERPESVIRUS‐1 (EHV‐1) OUTBREAKS FINDS NO EVIDENCE FOR A SIGNIFICANT EFFECT OF VACCINATION ON THE EFFECTIVE REPRODUCTION NUMBER

### **R.M.A.C. Houben** ^1^, C. Maanen^2^, J.R. Newton^3^, J. Broek^1^, M.M. Sloet^1^, J.A.P. Heesterbeek^1^

#### ^1^Utrecht University, Utrecht, The Netherlands; ^2^Royal GD, Deventer, The Netherlands; ^3^University of Cambridge, Cambridge, UK

**Introduction**: EHV‐1 infection is the cause of high impact disease syndromes, affecting the global horse industry. The effect of vaccination on transmission dynamics of EHV‐1 in naturally occurring outbreaks is insufficiently quantified. Our objective was to estimate *R*_0_ for EHV‐1 in equine herds from outbreak reports and evaluate the effect of herd vaccination status.

**Methods**: Systematic review, model‐based estimations, meta‐analysis. A literature search for outbreak reports was carried out. Depending on available data, the early epidemic growth rate (GR) or final attack rate (AR) approach was used to estimate the basic reproduction number for that outbreak. Herd vaccination status and strain type, and use of antivirals were also recorded. Only outbreaks in herds where either none or all horses had been vaccinated within 6 months before the outbreak were included. An overall estimate for *R*_0_ (non‐vaccinated herds) and *R*_*v*_ (vaccinated herds) was computed by meta‐analysis and the two groups were compared using a mixed effects model.

**Results**: Ten outbreaks met the inclusion criteria, of which five occurred in non‐vaccinated herds and five in vaccinated herds. One *R*_0_ calculation derived from a report describing empirical determination of a herd immunity threshold was also included. We did not detect a significant effect of vaccination status of the herd on the effective reproduction number in the included outbreaks: *R*_0_ = 3.3(2.6‐4.1) and *R*_*v*_ = 2.8(2.1‐3.5), *P* = .285. Insufficient (discordant) data were available to investigate the influence of strain type, vaccine type or antivirals on this result.

**Conclusions**: We were unable, with the available evidence, to support the assumption that herd vaccination significantly decreases transmission of EHV‐1. The lower limit of the *R*_*v*_ confidence interval was >1.

**Clinical Relevance**: Herd vaccination as a sole mitigating measure may have insufficient effect on transmission of EHV‐1 to prevent major outbreaks.

## EFFICACY OF AN ACTIVE VACCINATION AGAINST IL‐5 FOR THE TREATMENT OF EQUINE RECURRENT URTICARIA

### **K. Birkmann** ^1,2^, N. Waldern^1^, F. Jebbawi^1,3,4^, F. Canonica^1,3,4^, A. Fettelschoss‐Gabriel^1,3,4^

#### ^1^Evax AG, Im Binz 3, 8357 Guntershausen, Switzerland; ^2^Equine Clinic, Ludwig‐Maximilians University Munich, Sonnenstrasse 14, 85 764 Oberschleißheim, Germany; ^3^University Hospital Zurich, Department of Dermatology, Wagistrasse 18, 8952 Schlieren, Switzerland; ^4^Faculty of Medicine, University of Zurich, Switzerland

**Introduction**: Equine recurrent urticaria is a common clinical sign with various causes. Pathophysiology of equine urticaria is still poorly understood. However, a significant increase in inflammatory eosinophils in the dermis of lesional skin was confirmed. IL‐5 is the key‐cytokine for development, attraction and activation of eosinophils and might play a major role in the pathophysiology of equine recurrent urticaria. Therefore, the aim of our placebo‐controlled double blinded randomized study was to evaluate the efficacy of a previously described active vaccination against IL‐5 (eqIL‐5 vaccine) for the treatment of equine recurrent urticaria.

**Methods**: Exactly 36 client‐owned horses with recurrent urticaria were enrolled into the ongoing study. At screening visits, lesional and non‐lesional skin punch biopsies were collected for gene expression analysis by qPCR. Horses were randomly assigned to treatment or placebo group and received a basic vaccination either with the eIL‐5 vaccine or placebo. After completion of the basic vaccination, horses entered a blinded follow‐up, where all horses received the eqIL‐5 vaccine either as a re‐vaccination or as a basic vaccination. During both study phases, urticaria lesions were scored according to an urticaria activity score, every 4 weeks at live visits (UAS live), in weekly intervals from photographs (UAS photo). UAS scores of subgroups were compared by repeated measures ANOVA.

**Results**: Gene expression confirmed role of eosinophilic genes in lesional skin punch biopsies. An interim analysis showed statistically significant improvement of UAS live and photo scores in vaccinated horses.

**Conclusions**: These results suggest that IL‐5 plays a major role in the pathogenesis of equine recurrent urticaria and active vaccination against IL‐5 is an effective treatment.

**Clinical Relevance**: Since identification and elimination of the inciting antigen is often difficult and other treatment options carry the risk of severe side effects, this therapeutic vaccine targeting IL‐5 offers an effective treatment for equine recurrent urticaria.

Keywords: active vaccination, IL‐5, recurrent urticaria

## THE HYPOTHALAMIC‐PITUITARY‐ADRENAL GLAND AXIS RESPONSE TO VASOPRESSIN (AVP) STIMULATION TEST IN HEALTHY AND CRITICALLY ILL FOALS

### **K.A. Dembek**, E.M. Elder

#### North Carolina State University, Raleigh, USA

**Introduction**: Sepsis remains the leading cause of death in foals. The hypothalamic‐pituitary‐adrenal gland axis (HPAA) dysfunction is a common complication of sepsis resulting in decreased survival. HPAA dysfunction can be diagnosed with AVP stimulation test in other species. The goal of this study was to evaluate HPAA response to AVP stimulation in healthy and hospitalized foals. We hypothesized that AVP would stimulate a rise in ACTH and cortisol in healthy foals. We also proposed that cortisol and ACTH response would be decreased in critically ill foals compared to healthy foals, and that the diminished response would be associated with disease severity and outcome.

**Methods**: HPAA function was assessed in 12 healthy foals utilizing 2 doses of AVP (2.5, 5 IU), administered at 48 hours of age. Hospitalized foals (n = 18) were <7‐days old and received 2.5 or 5 IU of AVP on admission. Cortisol and ACTH were measured at 0, 15, 30, 60, and 90 minutes after AVP administration with immunoassays. A fold increase 15 and 30 minutes from baseline was calculated for cortisol and ACTH concentrations.

**Results**: All doses of AVP resulted in a significant increase in cortisol concentration and a dose‐dependent increase in ACTH concentration over time in both groups. ACTH and cortisol concentration increased 15 and 30 minutes after all doses of AVP compared to baseline in healthy and hospitalized foals (*P* < .01). Cortisol and ACTH response to AVP administration (2.5 and 5 IU) at 30 and 15 minutes was lower in critically ill foals compared to healthy foals suggesting HPAA dysfunction (*P* < .05).

**Discussion**: Administration of AVP is safe and results in a significant rise in ACTH and cortisol in both healthy and hospitalized foals.

**Clinical Relevance**: A stimulation test with 2.5 and 5 IU of AVP can be considered for HPAA assessment in critically ill foals.

## INCREASED SERUM THYMIDINE KINASE 1 ACTIVITY IS MOSTLY NOT DIAGNOSTIC FOR EQUINE MALIGNANT LYMPHOMA

### J. Zeppenfeld^1^, J. Marchewski^2^, A. Gläsel^3^, **K.A. Roscher** ^1^

#### ^1^Equine Clinic, Internal Medicine, Giessen, Germany; ^2^Institute of Veterinary Pathology, Giessen, Germany; ^3^Clinical Pathophysiology and Clinical Pathology, Giessen, Germany

**Introduction**: Lymphomas are the most common malignant hematopoietic neoplasia in horses, but antemortem diagnosis is often challenging for clinicians. Measurement of serum thymidine kinase 1 (sTK1) activity as an inexpensive and almost non‐invasive biomarker is described in the literature revealing controversial results. The aim of the study was to evaluate the performance of a commercial assay for the measurement of sTK1, in particular in patients whose cytology was non‐diagnostic for lymphoma.

**Methods**: Activity of sTK1 was measured in 19 equids with lymphoma, 11 equids with non‐lymphoid neoplasia, 15 equids with non‐neoplastic diseases and 27 clinical healthy equids. Seventeen lymphoma patients underwent at least one cytological examination antemortem (blood smear; aspirate of lymph node, bone marrow, spleen or abdominal tumor; abdominal or thoracic fluid). With the exception of the healthy control group all patients underwent a complete post‐mortem examination.

**Results**: Median (range) sTK1 activity was 2.50 (0.49‐382.0) U/L in lymphoma, 2.90 (0.49‐18.4) U/L in other neoplasia, 1.28 (0.49‐28.5) U/L in non‐neoplastic diseases and 0.63 (0.49‐1.95) U/L in controls. Only differences between control and lymphoma (*P* = .0002) and control and neoplasia (*P* = .024) achieved significance. Lymphoma was diagnosed antemortem by cytology in 12 equids. To distinguish lymphoma from all other patients, a maximum likelihood ratio of 6.42 could be achieved for sTK1 >23.45 U/L (sensitivity 10.53%, specificity 98.36%). However, only 2 horses with lymphoma reached these levels and both of them were diagnosed by antemortem cytology (blood smear and lymph node aspirate respectively).

**Conclusions**: Compared to healthy controls, sTK1 activity was increased in equids with lymphoma and non‐lymphoid neoplasia. However, elevated levels are usually not a definitive sign of lymphoma.

**Clinical Relevance**: The study highlights the value of cytological examination for the diagnosis of lymphoma in equids.

## EQUINE ASTHMA SEVERITY CORRELATES WITH NEUTROPHIL EXTRACELLULAR TRAPS IN BRONCHOALVEOLAR LAVAGE FLUID

### **L.K. Meiseberg**, J. Delarocque, R. Imker, M. Köckritz‐Blickwede, B. Ohnesorge, N. Buhr

#### University of Veterinary Medicine Hannover, Foundation, Hannover, Germany

**Introduction**: Neutrophil extracellular traps (NETs) are released by activated neutrophils and consist of extracellular chromatin‐histone‐components, granule proteins, enzymes, and anti‐microbial agents. They are described in equine and human asthma patients, where they can cause and feed‐forward inflammation. The aim of this study was to investigate clinical relevance of NETs in horses affected by equine asthma (EA).

**Methods**: Exactly 26 horses underwent a complete respiratory workup including exercise testing, arterial blood gas and bronchoalveolar lavage fluid (BALF) analysis. EA severity was determined based on the consensus statement criteria. NET‐activated cells in BALF were quantified using immunofluorescence confocal microscopy and blood‐derived neutrophil reactivity was assessed ex‐vivo by cathelicidin stimulation.

**Results**: EA was absent in 8 horses, mild in 4, moderate in 6 and severe in 8. Ordinal regression revealed a positive association between NET‐activated cells in BALF and blood‐derived neutrophil reactivity with EA severity (*P* = .009 and .02, respectively). As indicated by Welch's ANOVA, the latter was also associated with pO_2_ at rest (*F*[3, 11.894] = 8.365, *ω* ^2^ = 0.582, *P* = .003), which was significantly lower in horses with severe EA compared to all other groups (mean difference: −19 to −14 mmHg, *P* ≤ .031). Nevertheless, the negative correlation between NET‐activated cells in BALF and pO_2_ at rest was weak to moderate (*r* = −.37).

**Discussion**: Proportion of NET‐activated cells in BALF and peripheral neutrophil reactivity increased with asthma severity, indicating that EA may be associated with local as well as systemic immunologic changes.

**Clinical Relevance**: Increasing our understanding of EA pathophysiology may help to develop new therapeutic approaches. For example, NET‐formation inhibitors may be investigated. Moreover, quantification of blood‐derived neutrophil reactivity may be considered as screening tool to identify horses at risk for severe EA.

## A NEW SCORING SYSTEM TO EVALUATE MUCOSAL SURFACE PATHOLOGY IN EQUINE GLANDULAR GASTRIC DISEASE

### **V.H.L Scott**, D.G.M. Sutton, N.P. Evans, D.E.F. McKeegan

#### University of Glasgow, Glasgow, UK

**Introduction**: Both ordinal scale and descriptive systems have been reported for assessment of equine glandular gastric disease (EGGD). A new point scoring system (NPSS) is presented that aims to quantify specific features of glandular mucosal pathology and facilitate assessment of chronicity and healing.

**Methods**: Sequential gastroscopy videos were reviewed from Thoroughbreds (n = 26) diagnosed and treated for EGGD as part of a larger research study. Lesions were first (T_0_) categorized qualitatively as “mild,” “moderate,” or “severe” by specialists in internal medicine. The change in appearance of glandular lesions during 4 to 12 weeks of treatment was reviewed. NPSS was developed by assigning numerical values to lesion features of erythema, fibrin, and hemorrhage. Total NPSS score was scaled according to lesion surface area. The proportion contribution (*p̂*) of each feature score to NPSS was calculated and feature profiles were generated for disease severity. In horses with improving or deteriorating EGGD, changes in feature profiles were calculated.

**Results**: Features with the highest mean proportion in NPSS for mild, moderate, and severe EGGD at T_0_ were erythema (*p̂* = 0.45), fibrin (*p̂* = 0.4), and hemorrhage (*p̂* = 0.53), respectively. Modal category change for horses with worsening EGGD was mild to moderate. Within this group, increasing lesion fibrin was the greatest feature change (Δ*p̂* = 0.18) and a feature profile similar to moderate EGGD at T_0_ was observed. Modal category change for horses with improving EGGD was moderate to mild and decreased lesion hemorrhage was the greatest feature change (Δ*p̂* = −0.2); a feature profile different to mild EGGD at T_0_ was observed.

**Discussion and Clinical Relevance**: NPSS highlights quantitative variability in mucosal surface pathology for differing severities of EGGD. Horses with worsening lesions show progression of surface pathology consistent with spectrum of disease at initial diagnosis. Feature profiles for horses with improving lesions do not reflect a reverse sequential, facilitating assessment of healing stages.

## SECRETOMIC PROFILES TO DISTINGUISH ADULT HORSES WITH NON‐COMPLICATED ACUTE GASTRO‐INTESTINAL DISEASE FROM THOSE WITH SEPSIS

### A. Blangy‐Letheule^1^, M. Bouaud^1^, B. Lauzier^1^, **A.A. Leroux** ^4^, S. Bourgoin‐Voillard^2^, D. Habert^3^, J. Courty^2^, M. Seve^2^, B. Rozec^1^

#### ^1^Université de Nantes, CHU Nantes, CNRS, INSERM, l'institut du thorax, Nantes, France; ^2^Université Grenoble Alpes, TIMC, PROMETHEE Proteomic Platform, Grenoble, France; ^3^University of Paris‐Est Créteil (UPEC), Inserm U955, Equipe 21, UMR_S955, Créteil, France; ^4^Oniris, Nantes, France

**Introduction**: Sepsis is a life‐threatening organ dysfunction resulting from a deregulated host response to infection. Like humans, adult horses are prone to sepsis development secondary to gastro‐intestinal disease (GID). The secretome is a set of proteins secreted by a cell at a given time and under certain conditions. Easily accessible from plasma and analyzable by proteomic approach, it represents an opportunity to identify biomarkers in sepsis. This study aims to improve early detection and management of equine sepsis of digestive origin by identifying a combination of biomarkers that might help in clinical decision‐making.

**Methods**: Sample and clinical data of healthy horse (HH, n = 12), horses hospitalized for GID without signs of sepsis until discharge (n = 12) and horses with signs of sepsis (SH, n = 11) were collected upon admission to the equine emergency department and over the first 2 days of hospitalization. Samples were analyzed using large‐scale tandem mass spectrometry based on a label free quantification to allow identification of deregulated proteins (DEPs). The receiver operating characteristic curve (ROC) of the DEP were then analyzed with R software.

**Results**: Protein selection identified 469 proteins. The study of DEPs over time in the SH group compared with the HH and GID groups revealed 46 and 20 DEPs only upon admission compared with the HH and GID groups respectively. Among these proteins, 5 were common between HH vs SH and GID vs SH analysis. ROC curves were established for different protein combinations in order to characterize the pathophysiological signature of SH. A combination of 4 DEPs that distinguishes sepsis from GID with an area under the curve of 99.6% was identified.

**Clinical Relevance**: This study identified a promising combination of diagnostic biomarkers of sepsis. These data allow us to distinguish molecular alterations between these two conditions and may help delineate treatment strategies.

## A PROSPECTIVE, RANDOMIZED STUDY INTO THE COMPLICATIONS AND OUTCOMES OF UPPER OR LOWER EYELID SUBPALPEBRAL LAVAGE TREATMENT SYSTEMS IN 66 EQUINE EYES (2015‐2023)

### **A.E. Graham**, H. Carslake, F. Malalana

#### University of Liverpool, Neston, UK

**Introduction**: Subpalpebral lavage systems (SPLs) are commonly used in horses for administration of topical ophthalmic medication. Evidence for the optimal location is lacking. The aim of this prospective, randomized treatment trial was to compare the rate and type of complications of SPLs located in the central upper‐ compared to the medial lower eyelid in hospitalized patients.

**Methods**: Horses admitted for ophthalmic treatment using an SPL from February 2015 to March 2023 were included if the ocular pathology did not necessitate placement of the SPL in a specific location. A coin toss was used to randomly determine the SPL location. SPLs were monitored at least daily, and complications defined as major (displacement of the footplate from the fornix ± corneal ulceration; loss of footplate; eyelid infection/abscess formation) or minor (loss of suture/tape; palpebral cellulitis; leakage or tube rupture; loss of injection port; subcutaneous swelling/abscess at suture site).

**Results**: Sixty‐six SPLs in 65 horses were included, with 36 (54.5%) located in the upper‐, and 30 (45.5%) in the lower eyelid, for a median (IQR) duration of 10 (8‐16.2) days. Fifty‐nine complications occurred in 38/66 SPLs (57.6%) (32 upper eyelids (32/59; 54.2%) and 27 lower SPLs (27/59; 45.8%)). Major complications occurred in 2 lower SPLs (2/59; 3.4%) and 8 upper SPLs (8/59;13.6%). The most common major complication was displacement of the lavage footplate from the conjunctival fornix (6/66;9.1%). The most common minor complication was loss of the suture or butterfly tape (19/66;28.8%). Univariable and multivariable logistic regression failed to demonstrate an association between SPL location and any, or major complications.

**Discussion and Clinical Relevance**: The biggest limitation was small study size. Although not significant, results may suggest that although the upper eyelid is not significantly more likely to see complications, any complications may be more serious.

## SINGLE‐CELL TRANSCRIPTOME PROFILING OF BRONCHOALVEOLAR CELLS IDENTIFIES A TH17 SIGNATURE IN SEVERE EQUINE ASTHMA

### **S.E. Sage** ^1^, P. Nicholson^2^, L.M. Peters^1^, T. Leeb^1^, V. Jagannathan^1^, V. Gerber^1^

#### ^1^Vetsuisse Faculty, University of Bern, Bern, Switzerland; ^2^Next Generation Sequencing Platform, University of Bern, Bern, Switzerland

**Introduction**: Severe equine asthma (sEA) has been attributed in turn to a Th2, a Th1, a Th17, or a mixed immune response type. This conundrum stems in part from the current experimental and technological limitations. Using the cutting‐edge single‐cell mRNA sequencing (scRNA‐seq) technology, we profiled the transcriptome of equine bronchoalveolar lavage fluid (BALF) at cellular resolution to elucidate the underlying immune mechanisms of sEA.

**Methods**: Cryopreserved BALF cells from 11 Warmblood horses (5 control, 6 sEA) underwent droplet‐based scRNA‐seq. Horses were selected based on history, clinical score, and BALF cytology (neutrophils >10% for sEA). Data pre‐processing was performed with the Cell Ranger standard workflow and downstream analysis with the R package Seurat. Differential gene expression (DGE) analysis used the mixed model method Nebula.

**Results**: A total of 60 262 bronchoalveolar cells recovered after quality control and filtering could be grouped into six major cell types: B cells, T cells, monocytes‐macrophages, dendritic cells, neutrophils, and mast cells. With the exception of mast cells, all cell types displayed significant heterogeneity, with previously and newly described cell subtypes. Monocyte‐lymphocyte complexes were identified. We detected a strong Th17 signature in sEA, with upregulation of the B cell chemoattractant *CXCL13* in intermediate monocytes. The B cells were six times more abundant in sEA horses, with a lower fraction of switched plasma cells. Naive CD4+ T, Treg and γδT cells also presented Th17‐polarization, with upregulation of *IL‐17A*, *IL‐17F*, *IL‐21*, and *CCL20*. Several genes involved in T cell function were dysregulated. Neutrophils presented an enhanced capacity for migration and neutrophil extracellular trap formation.

**Discussion**: Single‐cell profiling of BALF cells supports a predominant Th17 immune response driven by monocyte and T cell gene dysregulation in neutrophilic sEA. The reproducibility of these results should be investigated in other breeds.

**Clinical Relevance**: The dysregulated genes identified with scRNA‐seq are potential biomarkers of sEA and promising therapeutic targets.

## IMMUNOHISTOCHEMICAL EXPRESSION OF CYCLOOXYGENASE‐2 (COX‐2) IN EQUINE MELANOMAS

### **J. Pimenta**, J. Prada, I. Pires, M. Cotovio

#### University of Trás‐os‐Montes e Alto Douro, Vila Real, Portugal

**Introduction**: Melanomas are one of the most common types of skin cancer in horses. These tumors present an uncommon benign behavior in comparison to other species, with low invasiveness and metastatic rates, but tumoral mass growth is usually a concern.

COX‐2 is related to pathologic conditions such as oncogenesis promoting neoplastic cell proliferation, invasion and metastasization, however studies regarding its expression in horse's tumors are scarce.

The aim of this study was to evaluate the immunohistochemical expression of COX‐2 in equine melanomas.

**Methods**: Exactly 38 equine melanomas were processed by immunohistochemistry to COX‐2 and classified by extension of labeled cells in (0) negative; (1) 1%‐5%; (2) 6%‐20%; (3) 21%‐50%; (4) >50% and intensity of labelling in (0) negative, (1) weak, (2) moderate, (3) strong. A final score was calculated by multiplying the extension by intensity of labelling with ≤6 being classified as weak and >6 as strong expression of COX‐2.

**Results**: Concerning final score, 27.8% of melanomas had high COX‐2 expression (>6) and 72.2% had low expression (≤6). Regarding extension of labelling 15.8% presented a score of 4; 50% of 3; 18.4% of 2; 5.3% of 1; and 10.5% of 0. Regarding intensity 21.1% were scored as 3; 28.2% as 2; 39.5% as 1; and 10.5% as 0.

**Discussion**: The overall low COX‐2 expression in equine melanomas is in accordance with biological behavior of these tumors. The low levels of COX‐2 may contribute for the typical mass growth of equine melanoma instead of contributing to invasiveness that is related to high COX‐2 levels.

**Clinical Relevance**: COX‐2 selective nonsteroidal anti‐inflammatory drugs could be a possible initial therapeutical approach for dermal melanomas/melanomatosis that are achieving concerning dimensions and invasiveness and that can make difficult a future surgical excision. NSAID will act by reducing the proliferation rates and thus the mass growth.

## DETERMINATION OF A MATHEMATICAL SCORE OF SURVIVAL IN NEWBORN FOALS: RETROSPECTIVE STUDY ON FOALS ADMITTED TO INTENSIVE CARE AT THE LYON EQUINE VET HOSPITAL (CLINÉQUINE) BETWEEN 2007 AND 2020

### **A.A. Amar**, A. Benamou‐Smith

#### VETAGROSUP Lyon, Marcy L'Etoile, France

**Introduction**: During the neonatal period, foals are very susceptible to disease. Their state of health can deteriorate suddenly, and appropriate medical care requires time‐consuming and expensive intensive care. This warrants an accurate assessment of the vital prognosis on admission based on clinical exam and ancillary tests, for ethical reasons as well as economical considerations.

**Methods**: During this retrospective study, the medical records of 226 foals <21 days old, for which the outcome “survival” or “non‐survival” was known, were examined. Twenty‐five variables including information from history, physical examination and laboratory findings were examined for their association with survival. Variables associated with survival were entered into a multivariable logistic regression model to determine which ones would be included in the survival score. Of these, 3 variables were retained in the final model.

**Results**: Fifteen mortality risk factors used as prognostic tools, collected at admission, have been identified as statistically significant. Factors in the final model included inability of the foal to stand, hematocrit, and neutrophils count. The highest (2.45) and the lowest (−2.25) scores represented 92% and 9% probability of survival, respectively. Sensitivity, specificity, positive and negative predictive values for the survival score were 83.7%, 63.6%, 79.4%, and 70%, respectively.

**Conclusions and Clinical Relevance**: This study provides strong correlations between mortality and a reasonable number of usual clinical and clinical pathology parameters, directly usable in the neonatal period. Based on our foal population presenting a wide array of affections and breeds, we show that a core list of parameters involved in usual neonate assessment allows us to provide useful prognostic values and the survival score established in our study can be easily implemented using data available at the admission. Further evaluations of this scoring system in a prospective study are needed.

## EFFECT OF DOPAMINE ON GLUCOSE‐STIMULATED INSULIN PRODUCTION IN THE EQUINE PANCREAS IN VITRO

### F. Li^1^, E. Green^1^, R. Spence^1^, M. Laat^1^, S. Bailey^2^, N. Galinelli^2^, P. Harris^3^, J. Sonntag^4^, T. Warnken^4^, **M. Sillence** ^1^

#### ^1^Queensland University of Technology, Brisbane, Australia; ^2^The University of Melbourne, Parkville, Australia; ^3^Waltham Petcare Science Institute, Melton Mowbray, UK; ^4^Boehringer Ingelheim Vetmedica GmbH, Ingelheim am Rhein, Germany

**Introduction**: Insulin dysregulation (ID), a key risk factor for hyperinsulinemia‐associated laminitis (HAL), is often seen in older *Equidae* with pituitary *pars intermedia* dysfunction (PPID), that show a decline in dopamine production. We have recently shown that pergolide can attenuate the insulin response to a glycemic meal in animals with concurrent PPID and ID, but the link between dopamine and insulin requires further investigation. In other species, insulin secretion can be decreased or increased, by activating D_2_ or D_3_ dopamine receptors, respectively. This study aimed to determine the dominant effect of dopamine on glucose‐stimulated insulin secretion in healthy equine pancreata in vitro.

**Methods**: Samples of pancreas were collected from 12 mixed‐breed horses slaughtered for human consumption. Pancreas explants (50‐100 mg) were incubated for 1 hour in Kreb's buffer containing either 2.5 mM glucose (low glucose control), or 10 mM glucose (high glucose) plus dopamine at 0, 0.1, 1, 10, or 100 μM. Insulin concentrations were measured in samples of the incubation medium, using an enzyme‐linked immunosorbent assay. The data were adjusted for tissue weight then analyzed using repeated measures ANOVA and Bonferroni's *t*‐test.

**Results**: Dopamine had a bi‐phasic effect on glucose‐stimulated insulin production (*P* < .05). Compared with the high glucose control, insulin output was doubled at 10 μM dopamine (*P* < .05), but no increase was seen at 100 μM dopamine.

**Conclusions**: Dopamine can augment glucose‐stimulated insulin production in isolated tissue from healthy horses, potentially acting via D_3_ receptors, but this effect is counteracted by high dopamine concentrations, suggesting the activation of D_2_ receptors.

**Clinical Relevance**: A better understanding of the interaction between dopamine and insulin production could lead to new insights into PPID and ID, and new approaches for the prevention of HAL.

## OBESITY AND ASSOCIATED METABOLIC DISEASE CONDITIONS IN CONNEMARA PONIES IN IRELAND

### **A. Al Ansari**, N. Walshe, E. Golding, V. Duggan

#### University College Dublin, Dublin 4, Ireland

**Introduction**: Equine obesity and insulin dysregulation (ID) are major risk factors associated with endocrinopathic laminitis. This study was conducted to investigate the prevalence of obesity, increased adiposity and associated endocrine/metabolic disease conditions in Connemara ponies in Ireland.

**Methods**: Registered Connemara ponies from Ireland, were recruited through public and veterinary social media posts. Ponies underwent a clinical exam and information on the management and clinical history was obtained via an owner questionnaire. Body condition score (BCS) was measured using the Henneke system; cresty neck score (CNS) and regionalized adiposity were recorded. Blood glucose, triglycerides and basal insulin concentration (BIC) were measured in all ponies and an oral sugar test (OST) was performed in 102 ponies. To differentiate Pituitary Pars‐intermedia dysfunction (PPID) as a cause of ID, plasma Adrenocorticotrophic hormone (ACTH) concentration was measured in ponies ≥10 years old.

**Results**: Exactly 287 ponies were included; 77 ponies (27%) had BCS ≥7, 74 (26%) had CNS ≥2.5, and 179 (62%) had regionalized adiposity. Owner reported history and/or clinical evidence of chronic laminitis found in 149 ponies (52%), with divergent rings observed in 130 ponies (45%). Twenty of 214 (9.3%) ponies ≥10 years old had plasma ACTH concentration above the seasonal reference range. Current ID was confirmed in 24% of 99 ponies in which OST results were available. Hypertriglyceridemia was observed in 15 (5%) ponies and hyperglycemia in 14 (4.9%) ponies. The multivariable model illustrated that the odds of showing ID‐OST were significantly associated with generalized obesity (*P* < .0001).

**Conclusions**: Obesity, increased adiposity, laminitis, and metabolic derangements are prevalent in this native Irish pony breed.

**Clinical Relevance**: ID and associated metabolic conditions are major risk factors for endocrinopathic laminitis in this breed; owners and vets should be alerted to these risks.

## BREATH CHARACTERISTICS AND ADVENTITIOUS LUNG SOUNDS IN HEALTHY AND ASTHMATIC HORSES: ASSESSMENT OF A NOVEL DIGITAL AUSCULTATION‐BASED METHOD IN EQUINE ASTHMA

### **E. Greim** ^1^, J. Naef^1^, S. Mainguy‐Seers^2^, J.‐P. Lavoie^2^, S. Sage^1^, G. Dolf^1^, V. Gerber^1^

#### ^1^Swiss Institute of Equine Medicine (ISME), Bern, Switzerland; ^2^Faculty of Veterinary Medicine, St‐Hyacinthe, Canada

**Introduction**: Thoracic auscultation is essential in the diagnostic work‐up of equine asthma (EA), but is limited by low sensitivity for transient or localized lung sounds, high subjectivity and the lack of standardized terminology.

**Methods**: This prospective multicenter case‐control study investigated normal and pathological breath sounds in clinically healthy horses (12 controls) and horses with mild‐moderate EA (12 mEA) and severe EA in remission (5 sEAr) or in exacerbation (5 sEAe) using a novel digital auscultation device. Group assignment was based on clinical and tracheal mucus scoring, bronchoalveolar lavage fluid cytology, and lung function testing. Each horse was auscultated in 11 locations simultaneously for 1 hour, resulting in 5′478 to 27′852 breath recordings per horse. Per recording, 100 breaths were randomly selected, visually and acoustically assessed, and blindly scored for breathing intensity (normal/increased/decreased), abnormal sounds (wheezes, crackles or rattles) and coughs.

**Results**: Most (85.9%) of analyzed breathes were good quality, allowing sound characterization. Cough episodes appeared as wide vertical bands with high intensity (>2000 Hz), wheezes as thin horizontal bands (200‐1000 Hz), and crackles and rattles as series of high‐frequency peaks. Preliminary analyses showed that pathological sounds were significantly more frequent in sEAe, but not in sEAr or mEA, when compared to the control. Wheezes were associated with clinical score and tracheal mucus score, while breathing intensity was associated with clinical score and BALF neutrophil percentage.

**Discussion**: While this pilot study demonstrated the capacity of a digital auscultation device to detect and quantify normal and abnormal respiratory sounds in horses, additional analyses on a larger sample are required to determine its ability to discriminate mildly asthmatic from healthy horses.

**Clinical Relevance**: This digital auscultation device may ultimately improve the diagnostic value of auscultation in horses. This is the first step towards developing a user‐friendly auscultation device with automated real‐time diagnostic feedback.

## NEONATAL PIROPLASMOSIS, AN UNDERESTIMATED PROBLEM?

### **L.M.H. Hermans** ^1^, A.L. Leblond^2^, A.J. Josson^3^, J.G. Gasco^3^, C.B. Bonsergent^4^, L.M. Malandrin^4^

#### ^1^Equine Department, VetAgro Sup, University of Lyon, Marcy l'étoile, France; ^2^UMR EPIA, INRAE, VetAgro Sup, University of Lyon, Marcy l'étoile, France; ^3^Equine Department, VetAgro Sup, University of Lyon, Marcy l'étoile, France; ^4^Oniris, INRAE, UMR BIOEPAR, Nantes, France

**Introduction**: Clinical cases of piroplasmosis have been reported rarely in neonates, resulting in jaundice. The question is to estimate the valuability of including piroplasmosis in the differential diagnosis of jaundice in the neonates. The objectives of our study were to estimate the frequency of vertical transmission of piroplasmosis from infected asymptomatic broodmares to their foals and observe the symptoms in positive newborns.

**Methods**: Mares spending more than 6 months/year on pasture, in their last trimester of gestation were included, as well as their foals of <72 hours of age and born in a box/paddock. Blood smears were evaluated, and nested PCR (nPCR) were performed on collected blood samples.

**Results**: Seventy‐one mares and their foals were included. Among the mares, a prevalence of 35.2% (25/71) and 2.8% (2/71) was detected by nPCR for *Theileria equi* and *Babesia caballi*, respectively. Blood smear evaluation of 61 samples revealed presence of *T. equi* in 6 smears (9.8%) and in none, B. *caballi*. No mare showed symptoms at the time of sampling. Among the foals born from an infected mare, a prevalence of 8% (2/25) and 0% (0/2) for *T. equi* and *B. caballi*, respectively, was detected by nPCR. Parasites were detectable in the blood smears of both infected foals. None of the infected foals had symptoms at the time of collection or in the following days.

**Discussion**: These results confirm that vertical transmission of *T. equi* from infected broodmares to their foals may occur with a low prevalence (8%). Foals born from asymptomatic mares did not show clinical signs, which is consistent with previous publications. Nevertheless, symptomatic piroplasmosis may be sporadically observed in this age group.

**Clinical Relevance**: Vertical transmission of piroplasmosis and correlation with symptoms in neonates may be of importance for breeders and warrants further research.

## SALMONELLA SHEDDING AMONG COLIC CASES PRESENTING TO AN EQUINE REFERRAL HOSPITAL IN QATAR

### S. Baillie^3^, T.F. Moreira Fernandes^1^, **M.R. Robin** ^1^, C. Jamieson^2^, B.U. Uberti^1^

#### ^1^Equine Veterinary Medical Center, Doha, Qatar; ^2^Purdue university, 625 Harrison Street, West Lafayette, IN, USA; ^3^EVMC, Doha, Qatar

**Introduction**: Sub‐clinical shedding of Salmonella is known to be a cause of disease outbreaks in equine hospitals. Consequences include increased patient morbidity and mortality, economic loss, reputation damage and, potentially, zoonotic infection. Horses with gastrointestinal disease are more likely to shed salmonella while hospitalized than horses with other types of illness and the rapid identification of infected horses can help reduce the spread of nosocomial infection.

**Methods**: A prospective observational study was carried out between June 2021 and May 2023. All horses presenting with colic had samples submitted for Salmonella PCR and culture within 24 hours of arrival at the hospital. Horses were then kept at an increased biosecurity level until results were available. Data were analyzed to identify any association with clinical pathology data on arrival.

**Results**: Exactly 321 horses were tested during the 2‐year period. Twenty‐four cases tested positive (7.5% prevalence). No clinically significant association with clinicopathological data was identified.

Of the positive cases, 18 were PCR and culture positive, 3 were PCR negative and culture positive, 2 were PCR positive and culture negative and 1 was PCR positive with no culture submitted.

Of the positive cases, 14/24 underwent surgery and 10/24 were treated medically. Of the negative cases, 111 /297 underwent surgery and 185/297 were treated medically.

**Conclusions**: The prevalence of detectable salmonella infection in horses presenting for colic was 7.5%.

**Clinical Relevance**: The data support the hospital's ongoing biosecurity protocols for colic patients, although no comparison can be drawn with non‐colic patients. There was no evidence of any additional clinical data that could be used to modify biosecurity protocol risk factors. The use of both PCR and culture to detect subclinical salmonella shedding should continue.

## EVALUATION OF SYMMETRIC (SDMA) AND ASYMMETRIC (ADMA) DIMETHYLARGININES IN HEALTHY AND IN SYSTEMIC INFLAMMATORY RESPONSE SYNDROME (SIRS) NEGATIVE OR POSITIVE COLIC HORSE

### **F. Bindi** ^1^, I. Nocera^2^, G. Vallesi^3^, V. Meucci^3^, F. Bonelli^3^, A. Spadari^4^, R. Rinnovati^4^, M. Sgorbini^3^

#### ^1^Department of Veterinary Sciences, University of Pisa, Pisa, Italy; ^2^Institute of Life Sciences, Sant'Anna School of Advanced Studies, Pisa, Italy; ^3^Department Veterinary Sciences, University of Pisa, Pisa, Italy; ^4^Department of Veterinary Medical Sciences, University of Bologna, Bologna, Italy

**Introduction**: The aim of this study was to compare plasmatic concentrations of ADMA and SDMA in healthy vs SIRS‐positive/negative colic horses over‐time and to evaluate the correlation between ADMA/SDMA and SIRS score to determine the effectiveness of these biomarkers (BIOs) for the evaluation of SIRS severity.

**Methods**: The study was approved by Ethical Committee (Pisa University 2825/14) and involved 2 veterinary teaching hospitals. A total of 66 horses were enrolled: 17/66 were healthy and 49/66 were colic horses. At admission (T0), and then after 24(T24), 48(T48), 72(T72), and 96(T96) h, each horse underwent a complete physical exam and SIRS score evaluation. Blood samples were collected once in healthy and at each sampling times for colic horses to assess SDMA and ADMA concentration using the Ivanova's method. Data distribution was analyzed using the Komolgorov‐Smirnov test. Kruskal‐Wallis and Dunn's multiple comparisons test were applied to verify differences for ADMA and SDMA between healthy and colic horses for all the sampling time. Correlation between SIRS score and ADMA/SDMA at T0 was assessed using Spearman test (*P* < .05).

**Results**: Of the 66 horses enrolled, 17/66 were healthy, 15/66 colic SIRS‐negative and 34/66 colic SIRS‐positive horses. Statistical differences were found (*P* < .0001) for SDMA between healthy vs SIRS‐negative (T24‐T96) or SIRS‐positive (T0‐T96) horses, while no differences were obtained for ADMA (*P* = .1487). No correlation was observed between SIRS score and SDMA at T0 (*P* = .584).

**Discussion**: SDMA shows potential as a biomarker for distinguishing between healthy horses and sick colic horses both SIRS‐negative and SIRS‐positive; ADMA might not demonstrate comparable discriminatory efficacy. However, the potential of SDMA as a biomarker for assessing the severity of SIRS has not yet been established.

**Clinical Relevance**: Although further research is needed to determine the diagnostic and prognostic role, our results suggest that SDMA might be a promising biomarker for equine colic research.

## EFFICACY OF INTRAMUSCULAR OMEPRAZOLE IN HORSES WITH ESGD AND EGGD, WITH OR WITHOUT IBD, PREVIOUSLY TREATED UNSUCCESSFULLY WITH ORAL OMEPRAZOLE

### **L.C. Kranenburg**, C. Ludin, R. Boom

#### Utrecht University, Utrecht, The Netherlands

**Introduction**: The aim of this study was to investigate the success rate of intramuscular omeprazole in horses with ESGD and EGGD, with and without concurrent IBD, that had been previously treated unsuccessfully.

**Materials and Methods**: Twenty horses received 20 mL long‐acting intramuscular omeprazole (IMOM), 4 times with a 7‐day interval, following unsuccessful treatment during 28 days with oral omeprazole, combined with sucralfate in case of EGGD. Of the 20 horses, 4 horses were diagnosed with ESGD, 15 horses were diagnosed with both ESGD and EGGD and one horse was diagnosed with EGGD. In 10 horses a duodenal biopsy was obtained, and a histological diagnosis of IBD was made in 9 cases.

**Results**: The ESGD lesions improved in 18/19 (94.7%) and healed in 11/19 (57.9%) cases. In case of EGGD 10/16 (62.5%) of the lesions improved and 8/16 (50.0%) of the lesions had resolved.

In horses with ESGD the lesion score after treatment was significantly lower than before treatment, both in horses with IBD (*P* = .022) and those without IBD (*P* = .005). For EGGD there was only a significant change in lesion score in the group without IBD (*P* = .018), but not in the group with IBD (*P* = .345). In one horse out of 9 with IBD the glandular lesions were more severe after the treatment, while none of the lesions deteriorated in the group without IBD.

**Discussion**: Superior results are reported for the treatment of EGUS with intramuscular omeprazole, compared to oral administration. Intramuscular omeprazole led to significantly decreased lesion scores for both ESGD and EGGD in horses without concurrent IBD, even when these lesions had not responded to previous treatment with oral omeprazole.

**Clinical Relevance**: The efficacy of IMOM is lower than previously reported when initial oral treatment was not successful. The concurrent presence of IBD negatively influences the healing of EGGD.

## EVALUATION OF A NEW SMARTPHONE‐BASED DIGITAL STETHOSCOPE FEATURING PHONOCARDIOGRAPHY AND ELECTROCARDIOGRAPHY IN ADULT HORSES

### **F. Bindi** ^1^, T. Vezzosi^2^, E. Zucca^3^, D. Caivano^4^, F. Freccero^5^, M. Sgorbini^2^

#### ^1^Department of Veterinary Sciences, University of Pisa, Pisa, Italy; ^2^Department Veterinary Sciences, University of Pisa, Pisa, Italy; ^3^Department of Veterinary Clinical Sciences, University of Milan, Milano, Italy; ^4^Department of Veterinary Medicine, University of Perugia, Perugia, Italy; ^5^Department of Veterinary Medical Sciences, University of Bologna, Bologna, Italy

**Introduction**: The aim was to evaluate a novel smartphone‐based digital stethoscope (DS) designed for simultaneous auscultation and recording of phonocardiogram and one‐lead ECG in horses.

**Methods**: This prospective, multicenter study, approved by the Ethical Committee (Pisa University‐3/22), included 94 adult horses. Each animal underwent conventional auscultation with an acoustic stethoscope, phonocardiographic recordings, standard base‐apex ECG, and recordings with the DS. The audio and phonocardiographic recordings, standard and DS ECG traces were blind reviewed. Cohen's κ was used to calculate the agreement between conventional auscultation and standard ECG vs DS. The Cohen's kappa coefficient was interpreted as follows: ≤0 indicated no agreement, 0.01 to 0.40 slight, 0.41 to 0.60 moderate, 0.61 to 0.80 substantial, and 0.81 to 1.00 optimal agreement. The agreement between standard ECG and DS ECG tracings was assessed using the Bland Altman plot.

**Results**: Twenty‐one/94 had a heart murmur (13 systolic; 8 diastolic), 16/94 sinoatrial or second‐degree atrioventricular block, 7/94 atrial fibrillation and 3/94 premature complexes; in 7/94 horses, both arrhythmias and murmurs were present; 44/94 were healthy horses. All the audio recordings were considered interpretable and optimal agreement in the diagnosis of heart murmurs (*k* = 1) and arrhythmias (*k* = 0.98) was found between conventional auscultation and DS. A suboptimal (*k* = 0.68) and moderate (*k* = 0.48) agreement was observed for P and QRS polarity. All the ECG traces recorded with the DS were deemed interpretable. The bias (95% limits of agreement) between standard ECG and DS was 0.08 (−4.34 to 4.51 bpm) for heart rate, 0.02 (−0.01 to 0.05 seconds) for P wave, −0.37 (−7.40 to 6.66 seconds) for PR, 0.008 (−0.03 to 0.04 seconds) for QRS duration, −0.02 (−0.22 to 0.18 seconds) for QT, −0.005 (−2.23 to 2.22 seconds) for artifacts durations.

**Discussion**: The DS exhibited good feasibility and diagnostic accuracy in detecting both heart murmurs and arrhythmias in adult horses.

**Clinical Relevance**: The DS could be a useful device for equine cardiac screening, especially in field conditions.

## PREVALENCE AND ROLE OF GLUTEN INTOLERANCE IN 52 HORSES SUSPECTED OF INFLAMMATORY BOWEL DISEASE

### **L.C. Kranenburg**, C.L. Tijssen, A.J. Brom‐Spierenburg, R. Boom

#### Utrecht University, Utrecht, The Netherlands

**Introduction**: Food allergies have been suggested to play a role in the pathogenesis of inflammatory bowel disease (IBD). The aim of this study was to investigate the prevalence of gluten intolerance and its role in IBD in horses.

**Materials and Methods**: The serum of 52 horses with a histological diagnosis of IBD was tested for the presence of antibodies against transglutaminase‐2 (TGA). The test was considered negative in the case of a TGA IgA‐titer lower than 35 AU/mL, dubious between 35 and 55 AU/mL and positive above 55 AU/mL. Follow‐up data regarding outcome, the effect of a gluten free diet/management and general well‐being were obtained for horses with a positive or dubious TGA titer, 6 months to 6 years after the serological test.

**Results**: A positive result was obtained in 7/52 (13.5%) horses, a dubious result in 13/52 (25%) horses and a negative result in 31/52 (59.6%) horses. Follow‐up data were available for 14 horses, 6 with a positive test result and 8 with a negative result. Of those 14 horses, 3 had been euthanized for reasons related to IBD and 2 because of a strangulating intestinal lesion. For 12/14 (85.7%) horses the owner considered a gluten free diet/management to be of benefit to the horse and the clinical signs improved: appetite increased, abdominal discomfort and sensitivity were reduced, performance improved and fecal consistency improved. In some cases, all clinical signs disappeared. The diet had no noticeable beneficial effect in one horse and a questionable effect in another horse.

**Discussion**: A histological diagnosis of IBD may be associated with a positive or dubious gluten serology result and gluten intolerance can play a role in some cases of IBD. A gluten free diet and management appear to be beneficial.

**Clinical Relevance**: Testing for gluten intolerance can be relevant in managing IBD cases.

## THE EFFECTS OF DETOMIDINE INFUSION WITH AND WITHOUT VATINOXAN ON BLOOD GLUCOSE AND INSULIN CONCENTRATIONS IN HORSES

### **N. Jantunen**, M. Raekallio, H. Tapio, B. Obrochta, L. Gracia Calvo, R. Rivera Pöyhönen, K. Hagman, N. Karikoski

#### University of Helsinki, Helsinki, Finland

**Introduction**: Alpha‐2 adrenoceptor agonists are widely used for equine anesthesia regardless of their side effects. The aim of this study was to investigate the effects of detomidine infusion with and without a peripherally acting alpha‐2 adrenoceptor antagonist, vatinoxan, on blood glucose (BG) and insulin concentrations.

**Methods**: Eight Finnhorses were assigned to two 4‐hour infusions: detomidine (0.01 mg/kg + 0.015 mg/kg/h IV) (DET) and a combination of DET and vatinoxan (0.15 mg/kg + 0.05 mg/kg/h IV) (DET + VAT) using cross‐over design. Blood samples were taken before, during and for 4 hours after the infusion at 1 hour interval. Blood glucose was analyzed with a portable glucometer (AlphaTRAK2) and serum insulin concentration with ELISA (Mercodia equine insulin ELISA).

**Results**: Mean BG peaked (17.0 ± 2.2 mmol/L) at the end of DET infusion and was higher than with DET + VAT (10.0 ± 1.6 mmol/L, *P* < .001). During DET infusion, median insulin concentration was lower than limit of quantification (1.15 μIU/mL, min‐max <1.15‐1.49 μIU/mL) and lower than with DET + VAT (2.24 μIU/mL, min‐max <1.15‐4.58 μIU/mL, *P* = .018). With neither treatment, insulin concentration reached the baseline within 4 hours after the infusion.

**Discussion**: Vatinoxan alleviated the detomidine‐induced increase in BG and decrease in serum insulin concentration during 4‐hour infusion and may be a beneficial addition to equine anesthesia protocols.

**Clinical Relevance**: Perioperative hyperglycemia is known to be associated with adverse events in humans but the role of transient hypoinsulinemia is less well recognized. In horses, both phenomena warrant more research.

## URETEROLITHIASIS IN EQUIDS: A RETROSPECTIVE STUDY OF 7 CASES (2013‐2022)

### **D. Jean**, L. Kamus, G. Fouquet, P. Ruzickova

#### University of Montreal, St‐Hyacinthe, Canada

**Introduction**: Ureterolithiasis in equids is poorly documented and usually associated with a low survival rate. The objectives are to describe the clinical findings, alterations in renal function and short‐term survival rate of equids with ureterolithiasis.

**Methods**: Medical files from 2013 to 2022 with ureterolithiasis as a primary or secondary diagnosis in seven equids presented to the Centre Hospitalier Universitaire Vétérinaire (CHUV) were reviewed. Follow‐up was obtained by phone call.

**Results**: Seven equids (6 horses of various breeds and one donkey including 6 geldings and one mare) between 11 and 36 years old were included. Urethral calculi were removed digitally via the urethral orifice (4 cases), surgically via urethrotomy (2 cases) or not removed (1 case). The short‐term survival rate was 100% with a hospitalization duration from 3 to 42 days. Hypercreatininemia (recorded highest value during hospitalization ranging from 134 to 924 μmol/L) was observed in 6/7 patients and renal function was considered normalized in 4/7 patients at discharge. Ultrasonographic evaluation showed anomalies of kidneys in all patients including nephromegaly (4 cases), renal pelvis dilation (5 cases) and abnormal corticomedullary junction definition (3 cases). Cystoscopy revealed: urethritis and cystitis (7 cases), ureteral opening abnormalities with edema and dilation (5 cases), sabulous urolithiasis bladder (3 cases) and, bladder necrosis (2 cases) including one case resulting in a rupture. Urolithiasis was identified in several anatomic location in 5/7 patients, including bladder (3 cases) and kidneys (5 cases). Follow up was documented for 4 patients (1‐5 years post discharge) without recurrence and 2/4 patient showing signs of chronic renal disease.

**Discussion and Clinical Relevance**: In the present study, equids with ureterolithiasis had a better short‐term survival rate than previously described in the literature. Improved survival was described with or without altered renal parameters, or ultrasonographic changes in the renal parenchyma.

## CLINICAL FINDINGS OF EOSINOPHILIC KERATOCONJUNCTIVITIS IN HORSES

### **Z. Bakos** ^1^, R. Lorenz^1^, J. Toth^2^

#### ^1^University of Veterinary Medicine Budapest, Budapest, Hungary; ^2^Tierärztliches Kompetenzzentrum Karthaus GmbH, Dülmen, Germany

**Introduction**: Eosinophilic keratoconjunctivitis (EKC) is an immune‐mediated disease of the cornea and conjunctiva of the equine eye. The aim of the study was to analyze the clinical records of horses diagnosed with EKC between 2012 and 2020 at a private equine hospital in Germany.

**Methods**: Medical records of 114 horses were collected. Retrospective data analysis included age, breed, gender, year and month of admission, affected part of the eye, clinical signs, laboratory results, presence of secondary infections, treatment, recovery time, and recurrences.

**Results**: Warmbloods were overrepresented (79%). The disease showed seasonality, the highest number of cases were diagnosed in July, August, and September. Based on the appearance of the lesions, a new classification has been suggested consisting of granulomatous, ulcerative, diphtheroid/pseudomembranous, and mixed forms of EKC. Only the cornea was affected in 59 patients (51.8%). Only the conjunctiva was involved in 17 cases (14.9%), and 33 horses (28.9%) showed lesions on both the cornea and the conjunctiva. Five horses (4.4%) had lesions on the cornea and additionally on the third eyelid. The disease was bilateral in 43% of the cases. Treatment regimens were chosen individually, and in 90 horses (78.9%) it was multimodal including laser therapy, diamond burr debridement and keratectomy besides medical therapy. Recovery time was extremely variable ranging from 5 to 330 days (median ± interquartile range: 30 ± 39 days). The affected eye was surgically removed in 8 horses (7%). Recurrence occurred in two horses (1.8%).

**Conclusions**: Our results demonstrate that EKC is an emerging ocular disease with variable clinical manifestations. Successful treatment often requires individualization, and a multimodal approach.

**Clinical Relevance**: To our knowledge, the current study population is the largest in the literature, thus our results may provide a deeper insight into this ocular condition to better understand its clinical features.

## THE EFFECT OF HIGH‐CARBOHYDRATE FEEDING AND BODY CONDITION ON PANCREATIC HISTOMORPHOMETRY IN MIXED‐BREED PONIES

### **K. Timko** ^1^, M. Watts^2^, J. McCutcheon, P. Weber, G. Ray, J. Belknap, T. Burns

#### ^1^The Ohio State University, Columbus, USA; ^2^The Ohio State University, 614‐292‐6661, USA

**Introduction**: Hyperinsulinemia‐associated laminitis is a debilitating disease that affects many equids. The endocrine pancreas is the source of endogenous insulin and presumed to be dysfunctional in hyperinsulinemic animals. The objective of this study was to assess pancreatic insulin, glucagon, and somatostatin expression in ponies fed a high non‐structural carbohydrate (NSC) diet.

**Methods**: Twenty‐one adult mixed‐breed ponies were divided into four experimental groups based on body condition scoring (lean: BCS4, obese: BCS7) and diet (low NSC [1.8 g/kg/day NSC], high NSC [8 g/kg/day NSC]): lean, low‐NSC (n = 5); obese, low‐NSC (n = 5); lean, high‐NSC (n = 5), and obese, high‐NSC (n = 6). Ponies received their respective diets for 7 days. Pancreas samples were collected following the feeding protocol and evaluated for immunohistochemical expression of insulin, glucagon, and somatostatin within islets. Insulin and glucose dynamics in this population were previously evaluated.

**Results**: The mean percent surface area of pancreatic islet cells expressing insulin, glucagon, and somatostatin in ponies fed the low‐NSC diet were 63%, 21%, and 5%, respectively, and 50%, 20%, and 3% in ponies fed the high‐NSC diet. Insulin and somatostatin expression were decreased in ponies fed the high‐NSC diet compared to low‐NSC diet (*P* = .044, 0.046), whereas glucagon expression did not differ. There was a negative correlation with somatostatin expression and serum [insulin] (*P* = .018, *r* = −.55), but no correlations with insulin or glucagon expression were observed. There were no differences among groups associated with BCS.

**Conclusions**: Insulin and somatostatin expression were reduced in ponies fed a high‐NSC diet, indicating a reduction in ‐cell and ‐cell surface area, respectively.

**Clinical Relevance**: Ponies fed a high‐NSC diet for 7 days demonstrate altered pancreatic histomorphology. Somatostatin expression was lower, which may contribute to hyperinsulinemia. A reduction in pancreatic insulin expression despite elevated serum [insulin] suggests the possibility of reduced insulin clearance.

## CONTACT FORCE‐GUIDED 3D ELECTRO‐ANATOMICAL MAPPING AND RADIOFREQUENCY ABLATION (CARTO3) FOR IMPROVED DIAGNOSIS AND TREATMENT OF SUSTAINED ATRIAL TACHYCARDIA IN 9 HORSES

### **E. Buschmann**, G. Van Steenkiste, I. Vernemmen, M. Demeyere, S. Schauvliege, A. Decloedt, G. Loon

#### Ghent University, Merelbeke, Belgium

**Introduction**: Treatment of atrial tachycardia by three‐dimensional electro‐anatomical mapping (3D EAM) and radiofrequency catheter ablation (RFCA) has been described using a non‐contact force system, but recurrence was still seen in some patients. This could be caused by inadequate catheter‐tissue contact during RFCA, resulting in incomplete ablation lesions. Real‐time assessment of the contact force (CF) between catheter and tissue might improve procedural success and decrease arrhythmia recurrence rate. Contact force‐guided ablation has not yet been used to treat arrhythmias in clinical patients.

**Methods**: Records from nine horses with sustained atrial tachycardia treated by RFCA, using a CF‐guided mapping and ablation system (CARTO3), were reviewed.

**Results**: The 3D EAM of the right atrium was performed in a mean time of 50 ± 27 minutes and revealed a clockwise re‐entry (n = 6), a counter clockwise re‐entry (n = 2) and a focal source (n = 1), all located in the caudomedial aspect of the right atrium. Point‐by‐point RFCA was performed in power‐controlled mode, with a mean of 18 ± 8 applications in 41 ± 16 minutes. A median power of 35[24‐45]W for a median duration of 19[8‐45]s was delivered, with a median CF of 11[3‐49]g and irrigation rate of 30 mL/min. A median ablation index of 439[312‐1018] was reached. Sinus rhythm was restored in all nine horses. To date, 3 to 24 months post‐ablation, none of the horses showed recurrence.

**Discussion**: Contact force‐guided RFCA with the CARTO3 system was feasible and effective to permanently treat the cause of sustained atrial tachycardia in horses. CF monitoring ensured efficient lesion creation, thereby minimizing the risk of recurrence.

**Clinical Relevance**: Compared to atrial fibrillation, treatment of atrial tachycardia using quinidine sulfate or transvenous electrical cardioversion can be more difficult, with a higher recurrence rate. The 3D EAM and RFCA using real‐time CF measurement not only allows to restore normal sinus rhythm, it might also minimize the risk for recurrence of atrial tachycardia.

## DUST GENERATION AND MICROBIOLOGICAL AIR QUALITY WITH DIFFERENT BEDDING MATERIALS IN A HORSE STABLE

### **C.U.P. Herholz** ^1^, L. Wicki^1^, J. Siegwart^1^, P. Küng^2^, A. Burren^1^

#### ^1^Bern University of Applied Science, Zollikofen, Switzerland; ^2^MUUTU AG, Bern, Switzerland

**Introduction**: The stable climate is of paramount importance to the respiratory health of horses.

**Methods**: Four bedding materials (deep straw mattress, daily cleaned straw, dedusted soft wood granulate and bio‐compost) were compared with respect to the amount of airborne particular matter (PM2.5 and PM10) at two different heights in a horse box (50 and 120 cm from the ground) and to the microbiological air quality of the air as colony forming units per cubic meter, (CFU/m^3^) of total bacteria, mold spores, total actinomycetes and the proportion of thermophilic actinomycetes. The bedding materials were tested for 10 days each. Dust was recorded continuously with two SDS011 sensors and microbiological air sampling was performed on days 1, 5, and 10 using an air sampling system (MBASS30v3, Holbach GmbH, Germany). The air temperature and humidity as well as the work in the barn were considered in the statistical analysis (R Core Team 2019, level of significance *P* < .05).

**Results and Discussion**: The differences between the bedding materials, in terms of dust were considered significant (*P* < .001), except for PM10 between soft wood granulate and bio‐compost (*P* > 0.05). The soft wood granulate presented the lowest values in terms of total germs (average 3′552 CFU/m^3^), total actinomycetes (average 528 CFU/m^3^) and the proportion of thermophilic actinomycetes (average 176 CFU/m^3^). The lowest values in mold spores were found with the deep layer straw litter (average 1′776 CFU/m^3^). The highest values were found with bio‐compost for all types of germs (average: total germs 21′097 CFU/m^3^; mold spores 38′860 CFU/m^3^; total actinomycetes 4′440 CFU/m^3^, the proportion of thermophilic actinomycetes 777 CFU/m^3^). Airborne particular matter (PM 2.5 and PM 10) and microbiological air quality were not correlated (*r* = .01).

**Clinical Relevance**: The type and management of bedding influences microbiological air quality and thus lung health.

## ASSOCIATION BETWEEN FUNGAL DETECTION AND DIAGNOSIS OF MODERATE EQUINE ASTHMA (MEA) ACCORDING TO SAMPLING SITE AND METHODOLOGY

### **P Barbazanges** ^1^, A Couroucé^1^, L.C. Lemonnier^1^, G. Le Digarcher^1^, J.M. Cardwell^2^, M.P. Toquet^3^, E.A. Richard^3^

#### ^1^Oniris, Nantes, France; ^2^Royal Veterinary College, Hatfield, UK; ^3^LABEO, Saint‐Contest, France

**Introduction**: Poor agreements were previously described between tracheal wash (TW) and bronchoalveolar lavage fluid (BALF), as well as fungal detection by cytology and mycology culture. The link between moderate equine asthma (mEA) and detection of fungal elements in the airways remains controversial.

**Objectives**: To determine the prevalence of fungal detection in TW and BALF and its association with diagnosis of mEA.

**Methods**: Prospective study on 120 horses in active training or referred for respiratory disease. Horses were classified as “control” or “mEA” based on clinical examination, airway endoscopy and BALF. A sample was considered positive if at least one colony was identified by culture or at least one fungal element was observed on cytology.

**Results**: Respectively, 35 and 85 horses were classified as “control” and “mEA.” No significant difference was observed between groups for fungal detection by cytology, regardless the sampling site. Prevalence of positive mycology culture was significantly higher for TW (89.4%) and BALF (31.8%) of mEA horses compared to controls (respectively, 68.6% and 8.6%). Diagnosis of mEA was significantly associated with positive mycology culture on both TW (OR = 3.9) and BALF (OR = 5.0) Mycology culture on BALF exhibited high specificity (0.90) and high positive predictive value (0.91), unlike mycology culture on TW (respectively, 0.76 and 0.31).

**Conclusion and clinical importance**: Despite a significant association with asthma diagnosis, the high prevalence of fungal detection in TW of control horses precludes its clinical relevance.

However, positive mycology culture on BALF represents a significant risk‐factor of suffering mEA.

## IMPROVEMENT OF GASTRIC ULCER AND RIDDEN HORSE PAIN ETHOGRAM SCORES WITH DIET ADAPTATION IN SPORT HORSES

### **V. G. M. Pineau** ^1^, F. ter Woort^1^, F. Julien^2^, M. Vernant, S. Lambey, E. Y. van Erck

#### ^1^ESMP, Waterloo, Belgium; ^2^Lambey SA, Torpes, France

**Introduction**: Gastric ulcers are highly prevalent in sport horses and may lead to poor performance, changes in behavior and impact horse welfare. We wanted to assess whether sole dietary changes affect gastric health and pain ethogram scores in ridden horses.

**Methods**: Nine showjumpers trained at the same stable receiving a pelleted diet high in sugar and starch (>30%) were examined at T0 and after 12 weeks (T12) of changing to a cooked, muesli‐type low‐starch (11%) diet. At each examination, the horses underwent a filmed standardized exercise test (SET) with the same rider. A ridden pain score (RHpE, out of 24) was calculated by two blinded observers watching the videos. The day after the SET, horses underwent a gastroscopy and ulcers were blindly scored using a proprietary score (out of 11, hyperkeratosis on 3, squamous ulcers on 4 and glandular lesions on 4). No antiulcer medication was administrated, horses were housed on shavings and received free choice hay. Horses were checked monthly for lameness. Results were analyzed with Wilcoxon and Spearman tests.

**Results**: After 12 weeks of the low starch diet, there was a significant improvement of ulcer scores (4.62.5 at T0 vs 1.01.0 at T12, *P* = .006) and of the RhpE scores (6.92.9 at T0 vs 2.92.0 at T12, *P* = .009). Total ulcer scores and glandular disease scores were positively correlated with RhpE scores (respectively, *r* = .436, *P* = .07, and *r* = .564, *P* = .015). Heart rate and blood lactates measured during SET were not significantly different at T0 and T12.

**Conclusion**: There is a positive correlation between ulcer scores and pain scores in ridden horses. A low starch diet significantly reduces the incidence of gastric ulcers and associated pain score during riding in horses.

**Clinical Relevance**: It is possible to mitigate gastric ulcers and to increase the equine athlete comfort during riding with dietary adjustments.

## SERIAL INVESTIGATION OF SEROPREVALENCE AND FECAL SHEDDING OF LAWSONIA INTRACELLULARIS IN THOROUGHBRED FOALS IN THE FIRST YEAR OF LIFE

### **C. Ribonnet**, L. Palmer, S. Bate, M. Cubitt, C. Bolton, A. Foote, C. Mackenzie, E. Floyd

#### Rossdales Veterinary Surgeons, Newmarket, UK

**Introduction**: Equine Proliferative Enteropathy is an intestinal disease of young horses caused by *Lawsonia intracellularis*. Seroprevalence is documented in several countries. This is the first UK‐based study with the aim to report seroprevalence, timing of seroconversion, frequency of fecal shedding and relationship with clinicopathological findings in Thoroughbred foals during their first year of life.

**Methods**: Clinical examination, hematology, biochemistry, serum immunoperoxidase monolayer assay (IPMA) for analysis of antibodies against *L. intracellularis* and fecal PCR were recorded for each foal at 24 to 48 hours, 3, 6, 9 and 12 months of age. A blood sample was collected from the dams at timepoint 0 for IPMA analysis.

**Results**: Exactly 47 foals from 6 different farms were enrolled and 37 completed the entire study. At timepoint 0, 77% of mares and 64% of foals were seropositive. There was a linear relationship between mare and foal serum titers (*r* = .75; *P* ≤ .001) and foals with failure of passive transfer had a significantly lower *L.intracellularis* titer (*P* = .0004; 95% CI 29.5‐93.6). 8/37 foals (22%) did not seroconvert during the study period. Peak timing for seroconversion was between 6 and 9 months of age. At 9 months, seropositive foals had significantly lower serum albumin concentrations (*P* = .029, 95% CI 0.048‐1.029) than seronegative foals. Only 2 fecal PCR samples tested positive for *L.intracellularis*, and no clinical signs of disease were recognized in any foal.

**Discussion**: This study indicates common exposure to *L.intracellularis* in UK Thoroughbred stud farms. Results correlate with the previously described timeframe for bacterial exposure and the highest risk for development of clinical disease. Lower albumin levels in 9‐month‐old seropositive foals suggests potential presence of subclinical disease.

**Clinical Relevance**: This information can help optimize screening policies, targeted treatments and vaccination schemes. Further investigation is needed to evaluate the subclinical effects on foal growth, development and future sales value.

## EFFECTS OF TIGHT NOSEBANDS ON THE UPPER AIRWAYS OF HORSES

### D. Scholler^1^, A. May^2^

#### ^1^Equine Hospital of Ludwig Maximilians University, Munich, Germany, Oberschleissheim, Germany; ^2^Equine Hospital of Ludwig Maximilians University, Munich, Germany

**Introduction**: The public perception of animal welfare in equestrian sports depends on training methods and presentation of horses at equestrian events. In this context, the often very tightly buckled nosebands, which are intended to prevent the horse from opening its mouth in response to a hard hand impact, also attracted a lot of attention. Various studies have evaluated the impact of tight nosebands on so‐called stress parameters—whereas the situation inside the pharynx has not yet been further looked at. Therefore, the main aim of the study was to evaluate the response of the pharyngeal structures to tight versus loose nosebands using overground endoscopy.

**Methods**: In this study, 16 warmblood horses were ridden with loose and tight nosebands while an overground endoscope was inserted. The animal study was approved by the government of Bavaria, Germany (approval AZ ROB‐55.2‐2532.Vet_02‐21‐100). For video analysis, five freeze frames each were prepared and analyzed at the beginning of expiration phase at rest after and during maximum exercise. The pharyngeal diameter was measured using a ratio of epiglottic width and a perpendicular line to a fixed point at the dorsal nasopharyngeal wall. Other findings such as swallowing, pharyngeal collapse, soft palate movements and secretion were also evaluated.

**Results**: While the pharyngeal‐epiglottic‐ratio (PE) did not change significantly in horses ridden with loose vs tight nosebands, there was a significant increase in parameters associated with discomfort in the pharyngeal region, for example, accumulation of secretions (*P* = .001) and pharyngeal collapse (*P* = .04) in horses ridden with tight nosebands.

**Discussion/Clinical Relevance**: The results show that tight nosebands do not only cause stress reactions visible from the outside, but also contribute to discomfort and adverse reactions in the pharyngeal region. These results may provide objective evidence for future decisions of equestrian sports organizations concerning further regulations on nosebands.

## DYNAMICS OF TRAINING AND ACUTE EXERCISE‐INDUCED SHIFTS IN MUSCULAR GLUCOSE TRANSPORTER (GLUT) 4, 8 AND 12 EXPRESSION IN LOCOMOTION (M. VASTUS LATERALIS) VERSUS POSTURE MUSCLES (M. PECTORALIS PROFUNDUS) IN HEALTHY HORSES

### C. Vidal de Moreno de Vega^1^, D. Lemmens^1^, C. Constance^1^, **B. Boshuizen** ^2^, L. Mare^1^, L. Leybaert^3^, K. Goethals^1^, J.E. Oliveira^4^, G. Hosotani^4^, D. Deforce^3^, F. Nieuwerburgh^3^, L. Devisscher^3^, C.J.G. Delesalle^1^

#### ^1^Ghent University, Faculty of Veterinary Medicine, Ghent, Belgium; ^2^Ghent University, Faculty of Veterinary medicine, Ghent, Belgium; ^3^Ghent University, Ghent, Belgium; ^4^Cargill, Vilvoorde, Belgium

**Introduction**: Important changes in muscle GLUT protein expression are expected if glucose influx plays a pivotal role in fueling metabolic pathways in response to exercise. Our aim was to assess dynamics of equine muscle GLUT4, GLUT8 and GLUT12 protein expression in response to training and acute exercise.

**Methods**: Sixteen untrained Standardbred mares (3‐4 years) performed an incremental SET at the start and end of 8 weeks harness training. Pectoralis (PM) and vastus lateralis (VL) muscle biopsies were taken before and after each SET, providing rest and acute samples in untrained and trained conditions, using Western blot for GLUT quantification and Image Pro1 for blot analysis. Data were normalized against GAPDH. Data was not normally distributed (Shapiro‐Wilk's test) therefore non parametric tests were used. Basal GLUT‐levels were analyzed with the Wilcoxon matched‐pairs. The effect of acute exercise and training on GLUT expression was assessed using the Friedman test with post hoc Dunn's.

**Results**: Basal GLUT4 and GLUT12 protein expression was significantly higher in the VL compared to the PM (*P*_GLUT4_ = .031; *P*_GLUT12_ = .002). Training had no effect on rest GLUT4 expression, neither in the VL, nor the PM (*P*_VL_ > .999; *P*_PM_ > .999). However, acute exercise in trained condition decreased GLUT4 expression in the VL (*P* = .015). No GLUT8 expression changes were recorded. Training decreased GLUT12 in rest VL biopsies (*P* = .036). This decrease was even more prominent in the VL after acute exercise in the trained condition (*P*_VL_ = .003).

**Discussion**: The important GLUT12 downregulation, both in answer to training and acute exercise; the GLUT4 downregulation after acute exercise in trained horses; and the lack of GLUT8 changes in any of the studied conditions, question the importance of glucose in equine muscle metabolism.

**Clinical Relevance**: These findings encourage to further explore alternative fuel involvement in equine muscular energetics.

## NT‐proBNP AS A POTENTIAL BIOMARKER FOR DIAGNOSIS, PROGNOSIS AND MONITORING OF CARDIAC DISEASE IN HORSES

### **M. Demeyere**, A. Dufourni, G. Loon, A. Decloedt

#### Ghent University, Faculty of Veterinary Medicine, Merelbeke, Belgium

**Introduction**: N‐terminal pro‐brain natriuretic peptide (NT‐proBNP) is currently the biomarker of choice in human and canine cardiology for diagnosis, prognosis and follow‐up of cardiac disease. This preliminary study aimed to investigate whether NT‐proBNP can be used as a diagnostic marker for cardiac dilation in horses.

**Methods**: Serum samples were collected from 20 healthy horses and 19 horses with left atrial (LA) and/or left ventricular (LV) dilation, aged 14.0 ± 5.2 years. Cardiac dilation was diagnosed when 2D and M‐mode echocardiographic measurements exceeded the upper reference range. Horses with cardiac dilation showed a grade 2 to 5/6 systolic or diastolic heart murmur caused by mitral or aortic valve regurgitation (n = 16), or a ventricular septal defect (n = 3). Clinical signs of congestive heart failure were present in one horse. Healthy horses did not show a murmur >1/6. Serum was stored at −20°C until analysis. NT‐proBNP concentration was determined using the Horse NT‐proBNP ELISA kit MBS014699 (MyBiosource, Inc., San Diego, USA).

**Results**: NT‐proBNP concentrations were significantly higher in horses with cardiac dilation (n = 19) compared to healthy horses (n = 20) (72.7 ± 31.3 vs 51.9 ± 23.4 pg/mL, *P* = .023). NT‐proBNP concentrations were also significantly higher in horses with LV dilation (n = 16) compared to horses with cardiac disease without LV dilation and healthy horses (n = 23) (75.3 ± 32.3 vs 52.9 ± 23.2 pg/mL, *P* = .016). No significant difference in NT‐proBNP concentrations was found between horses with LA dilation (n = 15) and horses with cardiac disease without LA dilation and healthy horses (n = 24) (68.7 ± 33.9 vs 57.9 ± 25.6 pg/mL, *P* = .267).

**Conclusion**: NT‐proBNP concentration differed significantly between horses with and without LV dilation. Further research in a larger group of horses with cardiac disease is needed to establish cut‐off values for cardiac dilation in order to estimate long‐term prognosis.

**Clinical Relevance**: NT‐proBNP might have an added value as diagnostic biomarker in the follow‐up of horses with structural heart disease.

## BASELINING PHYSIOLOGICAL PARAMETERS IN POSTURE VERSUS LOCOMOTION MUSCLES ACROSS BREEDS TOWARDS TAILORED DIETARY AND TRAINING MANAGEMENT

### C. Vidal Moreno de Vega^1^, C. Delesalle^1^, C. De Meeûs d'Argenteuil^1^, B. Boshuizen^1^, **L. De Maré** ^1^, Y. Gansemans^2^, F. Van Nieuwerburgh^2^, D. Deforce^2^, K. Goethals^1^, W. De Spiegelaere^1^, L. Leybaert^3^, L. Verdegaal^4^

#### ^1^Faculty of Veterinary Medicine, Ghent University, Merelbeke, Belgium; ^2^Ghent University, Ghent, Belgium; ^3^Faculty of Medicine and Health Sciences, Ghent University, Ghent, Belgium; ^4^Roseworthy Campus, University of Adelaide, South Australia, Australia

**Introduction**: Translating the physiological meaning of specific morphophysiological properties of specific muscle groups to their individual metabolic blueprint contributes significantly to a better understanding of the complex adaptations of muscle tissue to stimuli. The aim of the study was to map out and compare the fiber type composition, fiber type and mean fiber cross‐sectional area (fCSA, mfCSA) and metabolic blueprint of three muscles in 3 different breeds.

**Methods**: Muscle biopsies (m. pectoralis (PM), m. vastus lateralis (VL) and m. semitendinosus (ST)) were harvested of 7 untrained Friesian horses, 12 Standardbred and 4 Warmblood mares. Immunohistochemistry was analyzed using Image Pro software. Untargeted metabolomics was performed on the VL and PM of Friesian and Warmblood horses and the VL of Standardbreds using UHPLC/MS/MS and GC/MS. Overall effect of breed on fiber type percentage and fCSA and mfCSA was tested with Kruskal‐Wallis. Breeds were compared two‐by‐two with Wilcoxon rank‐sum test, with Bonferroni correction. Spearman correlation explored the correlation between the breed‐metabolites and the morphometric muscle‐parameters.

**Results**: Standardbreds had a significantly higher proportion of type IIA fibers in the PM and VL (*P* = .0003) with a bigger mfCSA (*P* = .0017) in the VL when compared to Friesians. Friesians showed significantly more type IIX fibers (*P* = .0047) in their PM. No significant differences in fCSA were seen across breeds. The lipid and nucleotide superpathways were significantly more upregulated in Friesians. Standardbreds showed highly active xenobiotic pathways and within the lipid superpathway, long and very long chain acylcarnitine upregulation. Amino acid metabolism was similar, except for increased branched chain amino acid and aromatic amino acid sub‐pathways in Friesians. The carbohydrate, amino acid and nucleotide superpathways and carnitine metabolism showed higher activity in Warmbloods.

**Discussion**: Results show important muscular metabolic breed differences and specificities.

**Clinical Relevance**: Results provide an essential basis for formulating breed‐specific dietary and training protocols.

## EVALUATION OF THE DELTA NEUTROPHIL INDEX (DNI) IN EQUINE NEONATAL SEPSIS

### **N. Ellero**, F. Lunetta, S. Fasoli, A. Mannini, C. Castagnetti, J. Mariella, F. Dondi, F. Freccero

#### University of Bologna, Bologna, Italy

**Introduction**: Delta neutrophil index (DNI) represents the fraction of circulating immature granulocytes in peripheral blood automatically calculated by ADVIA‐series hematology analyzers and has been associated with sepsis in human neonates. This study aimed to describe the diagnostic and prognostic potential of DNI in equine neonatal sepsis.

**Methods**: All foals undergoing a complete physical and hematochemical evaluation at birth and during the first 5 days of life or, in association with blood culture sample collection, at hospital admission were reviewed. One‐hundred and sixteen foals less than 5d old were enrolled and divided into: healthy (H group; n = 17); septic (S group; n = 23), in the presence of both positive blood culture and SIRS; sick‐nonseptic (NS group; n = 76). DNI was calculated by ADVIA2120i hematology system as the leukocyte difference between myeloperoxidase channel and nuclear lobularity channel count. Differences in leukocytes, DNI, SAA, outcome and neutrophils changes between groups were analyzed with Mann‐Whitney test, while Spearman's correlation coefficient was evaluated among different variables.

**Results**: In group H, based on a general linear model, no time‐dependent changes in DNI were detected and no differences were found between the three groups. Leukocyte and neutrophil counts were lower (*P* < .001), while SAA concentration was higher (*P* < .001) in S group than in NS group. Overall in sick foals, DNI was able to discriminate between survivors (n = 69) and non‐survivors (n = 30; *P* = .01), was higher in foals with the presence of neutrophils toxic changes on blood smear evaluation than in foals without alterations (*P* = .03) and was weakly correlated with SAA concentration (*r* = .3; *P* = .003).

**Discussion**: DNI could anticipate the morphological evaluation of blood smears to detect the left shift, which, together with SAA concentration, is an indicator of inflammation.

**Clinical Relevance**: DNI deserves further insights for its prognostic potential and for leukogram interpretation in sick foals, which, if done manually, may be operator‐dependent and time‐consuming.

